# Live imaging of the extracellular matrix with a glycan-binding fluorophore

**DOI:** 10.1038/s41592-024-02590-2

**Published:** 2025-02-06

**Authors:** Antonio Fiore, Guoqiang Yu, Jason J. Northey, Ronak Patel, Thomas A. Ravenscroft, Richard Ikegami, Wiert Kolkman, Pratik Kumar, Tanya L. Dilan, Virginia M. S. Ruetten, Misha B. Ahrens, Hari Shroff, Shaohe Wang, Valerie M. Weaver, Kayvon Pedram

**Affiliations:** 1 Janelia Research Campus, Howard Hughes Medical Institute (HHMI), Ashburn, VA USA; 2 Center for Bioengineering and Tissue Regeneration, Department of Surgery, University of California, San Francisco (UCSF), San Francisco, CA USA

**Keywords:** Glycobiology, Fluorescence imaging, Fluorescent dyes

## Abstract

All multicellular systems produce and dynamically regulate extracellular matrices (ECMs) that play essential roles in both biochemical and mechanical signaling. Though the spatial arrangement of these extracellular assemblies is critical to their biological functions, visualization of ECM structure is challenging, in part because the biomolecules that compose the ECM are difficult to fluorescently label individually and collectively. Here, we present a cell-impermeable small-molecule fluorophore, termed Rhobo6, that turns on and red shifts upon reversible binding to glycans. Given that most ECM components are densely glycosylated, the dye enables wash-free visualization of ECM, in systems ranging from in vitro substrates to in vivo mouse mammary tumors. Relative to existing techniques, Rhobo6 provides a broad substrate profile, superior tissue penetration, non-perturbative labeling, and negligible photobleaching. This work establishes a straightforward method for imaging the distribution of ECM in live tissues and organisms, lowering barriers for investigation of extracellular biology.

## Abstract (web summary)

Rhobo6 is a cell-impermeable small-molecule fluorophore that displays reversible fluorogenic binding to glycans, making it a general, wash-free, and non-perturbative label for the extracellular matrix in living samples.

## Main

The term ‘extracellular matrix’ encompasses the glycocalyx, which is cell membrane tethered; the interstitial matrix, which permeates the spaces between cells; basement membranes, which delineate the boundaries of cell populations; and connective tissues, such as fascia, tendons, and ligaments. The ECM is therefore a multiscale and heterogeneous body-wide structure. Throughout the lifespan of an organism, extracellular matrices are actively remodeled by myriad cell types and drive both biochemical and mechanical signaling^1,2^. For example, local increases in ECM stiffness can pattern the global orientation of developing mammary epithelium^3^, mechanical compaction of the ECM is sufficient to drive folding in engineered tissues^4^, and aberrant cell-surface glycosylation can drive tumor immune evasion and metastasis^5^. In these examples, as well as many others, the three-dimensional arrangement of ECM biomolecules over time is critical to their individual activities and composite properties, driving a longstanding interest in imaging the ECM in live tissues^6–8^.

Existing methods to fluorescently label extracellular biomolecules with affinity reagents, genetic tags, and chemical labels, however, are challenging to apply in live tissues. Protein-based affinity reagents, such as antibodies and lectins, are severely limited by poor spatial diffusivity^9^. Viral delivery of high-molecular-weight ECM components tagged with fluorescent proteins can be challenging owing to viral-packaging constraints, and endogenous genetic tags require substantial optimization to avoid perturbation of extracellular assemblies that are necessary for developmental viability^10,11^. More broadly, antibodies and genetic tagging are typically used to visualize one or a few targets in a given sample, but are difficult to multiplex sufficiently to provide a comprehensive view of ECM structure, especially given that there are heterogeneities in ECM composition between the cells of a tissue, tissues of an organism, and organisms. These challenges are illustrated by the dominance of label-free approaches, such as second-harmonic generation microscopy^12^ for visualization of fibrillar collagen, as well as efforts to develop collagen-binding small-molecule fluorophores^13,14^.

Glycosylation is a feature shared by nearly all ECM components^15^. As such, glycan-directed strategies have the potential to enable visualization of ECM structure en masse. Glycan labeling techniques can broadly be categorized into two types: metabolic incorporation of unnatural sugars and chemoenzymatic labeling^16^. Metabolic labeling is routinely used for imaging the glycocalyx in cultured cell systems and ex vivo tissue models, but generally requires >24 h of incubation with metabolic labels and is dependent on sample-specific glycosylation pathways^17^. Chemoenzymatic labeling involves the addition of an enzyme to the sample and is therefore subject to the spatial diffusivity constraints of protein-based methods^18^. Sodium periodate oxidation followed by aniline-catalyzed oxime formation^19^, an older technique for installing fluorophores on glycans, is toxic to live samples, necessitating staining at 4 °C and subsequent fixation. In general, because extracellular spaces, and by extension the ECM, are not protected by cell membranes, methods that involve buffer exchanges are more likely to induce mechanical and mass-action-driven perturbations to extracellular structures.

**Fig. 1:**
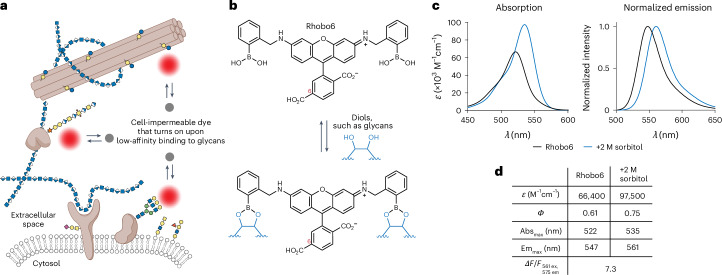
Photophysical characterization of the glycan-binding fluorophore Rhobo6. **a**, ECM labeling strategy. A cell-impermeable dye is added to a biological sample such that it disperses into extracellular spaces. Upon reversible association with glycoconjugates (colored shapes) of the extracellular matrix, the dye increases its fluorescence output. **b**, Rhobo6 structure and propensity for glycan binding. The carboxylic acid on the 6-position of Rhobo6 (red numbering) is charged at physiological pH, rendering the molecule cell-impermeable; the p*K*_a_ for *ortho*-aminomethylphenyl boronic acid is in the range of 5 to 7 (ref. ^31^), meaning the boronate and borate ester may dominate in aqueous buffer at physiological pH. **c**, Absorption and normalized emission spectra for Rhobo6 in unbound (5 µM dye in PBS) and bound (5 µM dye in PBS containing 2 M sorbitol) states. Emission spectra were measured with excitation wavelength at 490 nm. For 2P spectra, see Extended Data Figure 1i. *ε*, molar extinction. **d**, Table of photophysical properties. *ε* is reported at peak absorption. Quantum yield (*Φ*) is reported as the average value measured between 475 nm and 535 nm. Contrast is measured as the relative fluorescence signal change between bound and unbound states (Δ*F*/*F*), when exciting at 561 nm and detecting fluorescence signal at 575 nm. Because of red shift in both absorption and emission, the value is dependent on both excitation and emission parameters (see also Extended Data Fig. 1e–h). Abs_max_, wavelength of maximum absorption; Em_max_, wavelength of maximum emission. Source data

We envisioned labeling ECM architecture using a cell-impermeable small-molecule probe that increases its fluorescence upon reversible interaction with a chemical functionality commonly found on glycans (Fig. 1a). A small molecule would exhibit superior tissue penetration, and reversible, low-affinity binding would have the advantage of minimal perturbation to native structures and low photobleaching owing to an excess of unbound dye^20^. Further, by analogy to widely used DNA-minor-groove-binding fluorogenic small molecules (for example, Hoechst^21^), such a dye could be used as a one-step, wash-free dilution from a stock solution, and would be applicable to a wide range of sample types, including those that are not amenable to genetic manipulation and/or ex vivo culture. If successful, this method would lower barriers for testing hypotheses related to the composite properties of the ECM and, by extension, to biological phenomena in extracellular spaces.

Boronic acids have been known for decades to exhibit reversible binding to 1,2- and 1,3-diols, with dissociation constants in the tens of millimolar range^22^. Such diols are found in glycans and rarely elsewhere (for example, on the ribose at the 3′ end of RNA), to the extent that boronic acids are used for affinity purification of carbohydrates from complex biological samples^23^. In addition, boronic acids have a rich history of conjugation to fluorophores to yield ‘boronolectins’. This class of molecule, which includes boronated cyanines, rhodamines, BODIPY dyes, and others^24–26^, served as our starting point.

## Results

### Probe design and photophysical characterization

Of the previously described boronic-acid-dye scaffolds, we were drawn to a rhodamine-110-derived boronolectin, termed Rhobo, that had been developed by Strongin as a saccharide sensor for liquid chromatography^27,28^ and used by Schepartz to bind tetraserine motifs on peptides^29^. First, Rhobo contains a phenylboronic acid on each side of the xanthene core, increasing affinity for saccharides through avidity^30^. Second, Rhobo’s boronic acids are of the Wulff type, defined by the presence of an aminomethyl group *ortho* to the phenylboronic acid. Extensive studies by Anslyn, James, Shinkai, and Wang have shown that *ortho*-aminomethylphenyl boronic acids have the dual advantage of (1) lowering the p*K*_a_ of the boronic acids and thereby enhancing the thermodynamics of sugar binding at neutral pH, and (2) enhancing the kinetics of sugar binding through ammonium-mediated intramolecular general acid catalysis^31^. Additionally, fluorescence turn-on and spectral red shifts have been observed upon in vitro incubation of Rhobo with monosaccharides^28,29^.

**Extended Data Fig. 1:**
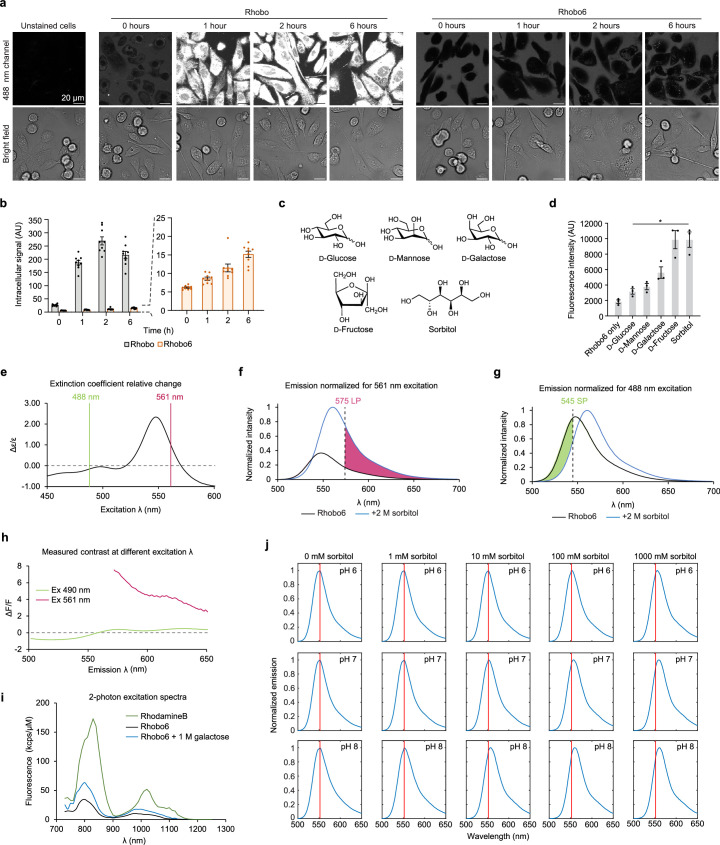
Rhobo6 cell impermeability and additional photophysical characterization. **a**, Comparison between Rhobo and Rhobo6 cell permeability over time. PC3 cells were incubated with Rhobo or Rhobo6 at 5 µM concentration in serum-containing media. Wells were then imaged directly after addition of dye (t = 0 h) and following a 1, 2, and 6 h incubation at 37 °C and 5% CO_2_. Cell surface signal is absent in this experiment due to excitation at 488 nm (cf. (**e**)-(**h**)) and the presence of serum-containing media (cf. Extended Data Fig. 3d). **b**, Quantified intracellular signal for Rhobo and Rhobo6 over time as determined by manually drawn regions of interest of *N* = 9 cells per condition. Error bars represent SEM. **c**, Monosaccharides and monosaccharide analogs used in (**d**). **d**, Quantified fluorescence of Rhobo6 measured by exciting at 555 nm and detecting fluorescence intensity maxima between 570 and 630 nm. All sugars were prepared in PBS solutions at 200 mM with 5 µM Rhobo6, pH 7.3-7.4, and incubated for 1 h at room temperature. *N* = 3, error bars represent SEM. *P* values were determined by unpaired two-tailed t-test with Welch’s correction, relative to dye only control; *P < 0.05. **e**, Relative change in extinction coefficient between bound and unbound Rhobo6 as a function of excitation wavelength calculated as (ε_bound_-ε_unbound_)/ε_unbound_. Position of standard laser lines 488 nm and 561 nm are shown to highlight that longer wavelength excitation preferentially excites bound dye, enhancing observed contrast. **f**, Emission spectra of bound and unbound Rhobo6 normalized for 561 nm excitation. A standard emission filter for red fluorescence (575 nm long pass [LP]) will preferentially collect light from bound dye, enhancing observed Contrast. **g**, Emission spectra of bound and unbound Rhobo6 normalized to 488 nm excitation (cf. Methods). A standard emission filter for green fluorescence (545 nm short pass [SP]) will preferentially collect light from unbound dye, reducing observed contrast. **h**, Measured contrast (calculated as ΔF/F between bound and unbound Rhobo6), as function of emission wavelength for 490 nm and 561 nm excitation. The plot agrees with renormalized spectral data in (**f**)-(**g**), with measured optimal contrast at 561 nm excitation and 570–580 nm emission. **i**, Two-photon excitation spectra for Rhobo6 in 100 mM phosphate buffer, pH 7.4 (unbound state) and 100 mM phosphate buffer containing 1 M galactose, pH 7.4 (bound state). The measured excitation spectrum of Rhodamine B is shown for reference. **j**, Normalized emission spectra of Rhobo6 incubated with various concentrations of sorbitol at pH 6, 7, and 8. At increasing pH, the concentration of sorbitol required to obtain a red-shifted population decreases, indicating increased Rhobo6 affinity towards sorbitol, as expected^31^. A vertical red line is shown at 552 nm, corresponding to the peak emission of unbound dye at each pH. Source data

Rhobo has been reported to rapidly cross cell membranes and label intracellular structures when applied at low micromolar concentrations to cells, which we confirmed using cultured cell monolayers (Extended Data Fig. 1a,b). Intracellular dye accumulation is unacceptable for imaging of ECM components, as it will (1) deplete dye from extracellular spaces and (2) substantially reduce the signal-to-background ratio in tissues.

The addition of functional groups that are charged at physiological pH is known to reduce cell permeability of small-molecule dyes (for example, calcein^32^). A cell-impermeable Rhobo analog was generated by adding a carboxylic acid substituent at the 6-position of the dye, which was achieved through a one-step reductive amination reaction with commercially available 6-carboxyrhodamine 110 and 2-formylphenylboronic acid (Methods) (Fig. 1b). The resulting molecule, which we term Rhobo6, exhibited dramatically reduced cell permeability relative to Rhobo during 6 h of incubation on cultured cell monolayers (Extended Data Fig. 1a,b).

Photophysical characterization of Rhobo6 in diol-free buffer revealed an approximately 20-nm shift in absorbance and emission maxima relative to those of the parent dye 6-carboxyrhodamine 110, closely matching reported values for dibenzylrhodamine (that is, a dye with *N*-benzyl groups lacking boronic acids)^33^. Next, a saturating concentration of the sugar alcohol sorbitol was added to generate the diol-bound form of the dye (Extended Data Fig. 1c,d). Relative to the unbound form, the bound form exhibited an increase in molar absorptivity, an increase in quantum yield, a 13-nm red shift in the absorbance peak, and a 14-nm red shift in the emission peak (Fig. 1c,d). Rhobo6 therefore turns on and red shifts upon binding diols. As a result of the red shift, the choice of excitation wavelength and emission filters will influence observed contrast (Extended Data Fig. 1e–h). Excitation with a 561-nm laser line coupled with a 575-nm longpass emission filter, corresponding to commonly used red fluorescent protein (RFP) imaging parameters, provided near-optimal fluorescence contrast, with a measured in vitro fluorescence change (∆*F*/*F*) of 7.3. The two-photon (2P) excitation spectra of Rhobo6 exhibited an 800-nm peak, which increased in the diol-bound state (Extended Data Fig. 1i). All measurements were taken at physiological pH. Of note, the affinity of Rhobo6 for diols increases with increasing pH, as is expected for phenylboronic acids (Extended Data Fig. 1j)^31^.

### Labeling profile for purified glycans and ECM components

**Extended Data Fig. 2:**
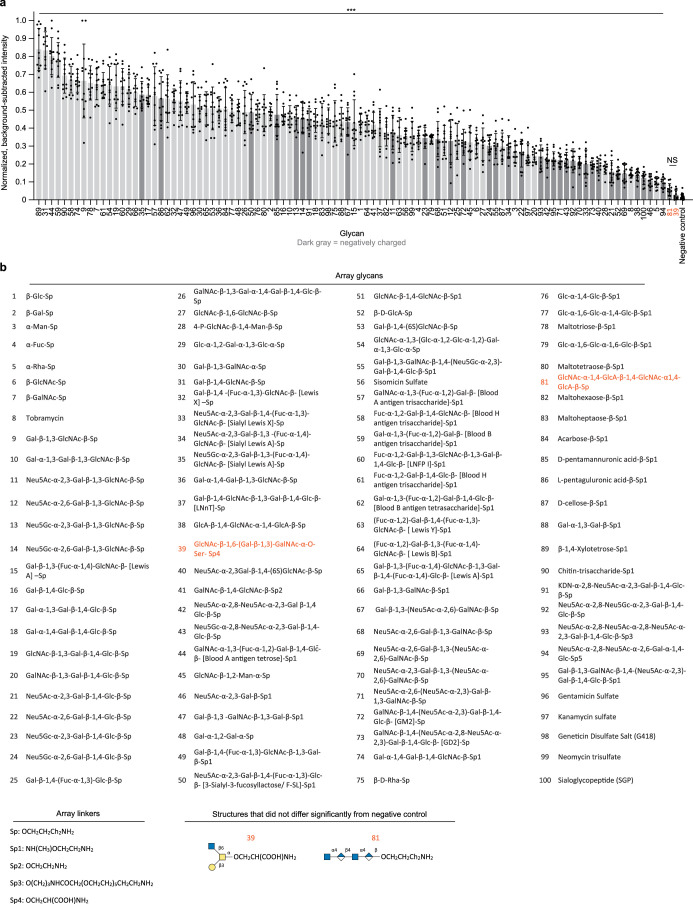
Application of Rhobo6 to a commercial glycan array. **a**, Normalized fluorescence signal of Rhobo6 measured by quantifying three glycan arrays with four glycan replicates each, printed on a single glass slide. Fluorescence signal was background corrected and normalized within each array. Negatively charged glycans are highlighted in a darker shade of gray. *N* = 12 for glycans, *N* = 24 for negative control, error bars represent standard deviation. Statistical significance was determined through unpaired two-tailed t-test with Welch’s correction, relative to the negative control group; NS = not significant (orange text), ***P < 0.0005. For all glycans not marked ‘NS’, average signal was confirmed to be greater than two times the standard deviation of local background. **b**, Array glycans and glycan linkers. Glycans marked ‘NS’ in (**a**) are colored orange. Note that linker ‘Sp4’ carries a carboxylic acid, which is negatively charged at physiological pH. Source data

To assess the specificity of Rhobo6 for glycans, a commercially printed glycan array was incubated with buffer containing 5 µM Rhobo6 and imaged without washing using a confocal microscope (Extended Data Fig. 2). Of the 100 glycans in the array, 98 showed a statistically significant increase in binding compared with negative controls, indicating broad specificity for glycans and glycoconjugates. The glycans with lower binding were enriched in negatively charged structures, suggesting that charge–charge repulsion could affect Rhobo6 binding.

**Fig. 2:**
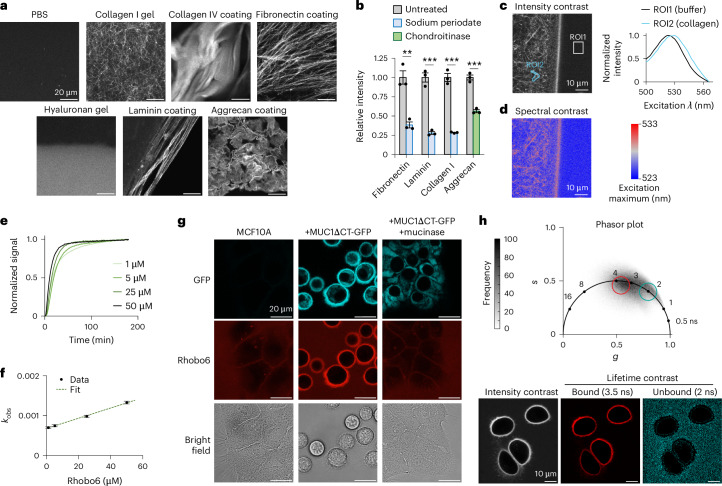
In vitro and in cellulo validation of Rhobo6 labeling. **a**, Rhobo6 labeling of purified ECM components. Substrates were prepared as glass coatings or gels (Methods), incubated with Rhobo6 at 5 µM for 1 h in PBS, and imaged using a confocal microscope. Contrast is not normalized across images. **b**, ECM components were treated with 10 mM sodium periodate (blue) or with chondroitinase ABC (green), and signal intensity relative to the untreated condition was quantified from confocal microscopy images. Periodate and chondroitinase treatment are expected to cleave a subset of glycans on the substrates shown. For representative images used for quantification, see Extended Data Figure 3a. *n* = 3; error bars represent s.e.m. *P* values were determined using an unpaired two-tailed *t*-test; **P < 0.005; ***P < 0.0005. **c**, Spectral imaging at the boundary of a collagen I gel and the surrounding buffer containing 5 µM Rhobo6, performed through an excitation scan (500–566 nm) and detection of fluorescence at 575–630 nm (Supplementary Table 1). The intensity contrast image (left) was obtained at 560 nm excitation with manually traced ROIs to capture an area rich in collagen fibers and an area in the surrounding buffer. Excitation spectra (right) correspond to the manually drawn ROIs. **d**, A spectral contrast image generated by plotting excitation maxima for each pixel in **c**. Two-pixel bins were used in the image. **e**, Time course of Rhobo6 fluorescence signal upon incubation with collagen I gels at varying concentrations. Binding curves were used to extract a value for *k*_obs_ at each concentration (Methods). **f**, The linear fit between *k*_obs_ and Rhobo6 concentrations from **e** allows extrapolation of the binding constants *k*_on_ and *k*_off_. An apparent *K*_d_ of 53 µM was determined by the ratio of the two. Error bars represent the 95% confidence interval for the fitted *k*_obs_ values. **g**, Confocal microscopy of MCF10A cells labeled with Rhobo6. Expression of GFP-MUC1∆CT was induced through the addition of doxycycline. Mucin domains, which are amino-terminal to GFP, were degraded enzymatically through live-cell treatment with the mucinase StcE^36^. Mucin overexpression in these cells induces membrane protrusions (villi), which are resolved as a thick halo around the cell; mucin overexpression also causes the cells to partially lift from their growth substrate, resulting in a spherical appearance with no loss in viability^35^. Contrast is normalized for each channel across experimental conditions. **h**, FLIM microscopy of MCF10A+GFP-MUC1∆CT cells labeled with Rhobo6. Top, phasor plot of lifetime distribution, with ROIs marking unbound and bound Rhobo6 populations. Bottom, intensity contrast image compared with lifetime bandpass images for each population (Supplementary Table 1). The broad distribution of observed lifetimes could be due to a multitude of possible binding modes, as well as variability in glycan structure. *g*, cosine transform; *s*, sine transform. Source data

**Extended Data Fig. 3:**
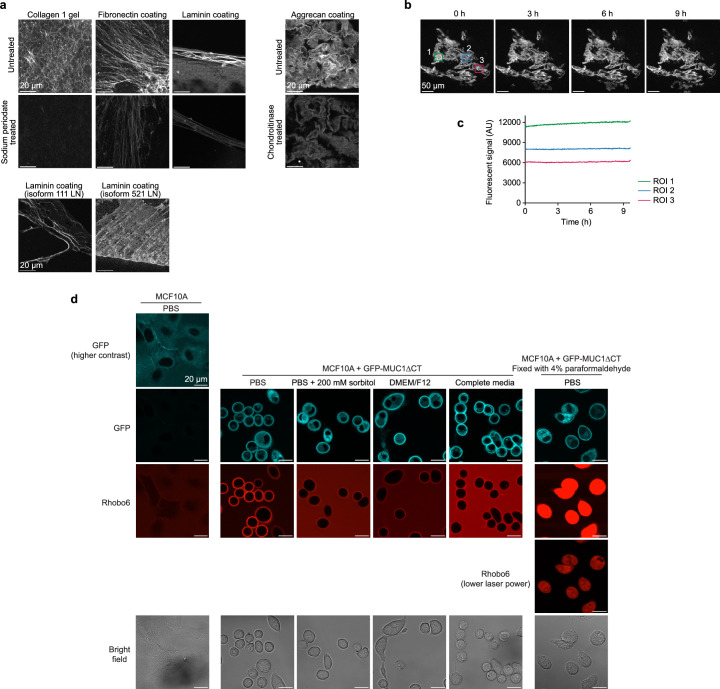
In vitro and in cellulo Rhobo6 characterization, related to Fig. 2. **a**, Representative fields of view from triplicates shown in Fig. 2b, along with additional Laminin isoforms 111 LN and 521 LN. Contrast is normalized within each untreated and treated substrate pair. Signal intensity is reduced upon treatment, as quantified in Fig. 2c. **b**, Frames extracted from 9.6 h imaging, at one frame per min, on a coated aggrecan substrate incubated with 5 µM Rhobo6. ROIs analyzed in (**c**) are highlighted in the first frame. **c**, Time trace of signal, calculated as mean intensity within three different manually traced ROIs, from (**b**). Photobleaching was not observed over the course of imaging. **d**, Effect of imaging buffer, mucin expression, and cell fixation on cell surface Rhobo6 signal. MCF10A + GFP-MUC1∆CT cells were incubated with doxycycline to induce GFP-tagged mucin expression, and imaged in different buffers containing 5 µM Rhobo6. PBS shows the best signal-to-background ratio. Cell surface signal can be partially competed away by addition of 200 mM sorbitol (cf. Extended Data Fig. 1d), confirming that Rhobo6 binding is diol-dependent and reversible. DMEM/F12 media contains 17.5 mM d-Glucose, likely contributing to a higher background than PBS. Supplementation of DMEM/F12 with the remaining complete media components (cf. Methods) results in the lowest observed signal-to-background ratio, likely due to the abundance of glycoconjugates in serum. MCF10A cells that were not induced to express GFP-MUC1∆CT were also imaged in PBS and exhibited dramatically lower cell surface Rhobo6 labeling, possibly due to the low density of binding sites on these cells. Finally, mucin-expressing cells were imaged after fixation with 4% paraformaldehyde; as a result of the fixation, membrane integrity is compromised, allowing Rhobo6 to accumulate in the cytosol. Rhobo6 is therefore not suitable for use with chemically fixed samples as it will accumulate non-specifically in fixed cells. Source data

Next, Rhobo6 was applied at 5 µM in phosphate-buffered saline (PBS) to purified ECM constituents, including fibrillar glycoproteins (collagen I, fibronectin), network-forming glycoproteins (collagen IV, laminins), a proteoglycan (aggrecan), and a polysaccharide (hyaluronan) (Fig. 2a and Extended Data Fig. 3a; images are not contrast normalized). Fluorescence contrast was observed across all substrates, with hyaluronan showing the weakest signal, possibly owing to a lack of condensed structures (see discussion of the dissociation constant (*K*_d_) below). Pretreatment with sodium periodate, which destroys 1,2-diols, reduced labeling of collagen I, laminin, and fibronectin, and pretreatment with the glycosidase chondroitinase reduced labeling of aggrecan, indicating that Rhobo6-mediated fluorescence contrast depends on the presence of glycans (Fig. 2b and Extended Data Fig. 3a). Spectral imaging of a collagen I gel incubated with 5 µM Rhobo6 showed a red shift in a region of interest (ROI) in the gel relative to an ROI in the buffer (Fig. 2c). Spatial mapping of the excitation maximum detected at each pixel yielded a spectral contrast image (Fig. 2d). Comparison of spectral- and intensity-contrasted images confirmed the presence of both free and bound Rhobo6 in the field of view, with collagen-bound dye molecules exhibiting a red-shifted excitation maximum.

We next estimated an apparent *K*_d_ for Rhobo6 binding to ECM substrates. The observed equilibrium constant (*k*_obs_) as a function of Rhobo6 concentration was measured using collagen I as a substrate (Fig. 2e)^34^. A linear fit allowed extrapolation of the association rate constant (*k*_on_) (12.8 M^−1^ s^−1^), dissociation rate constant (*k*_off_) (6.77 × 10^−4^ s^−1^), and *K*_d_ (53 µM) (Fig. 2f). The apparent *K*_d_ value that we measured is roughly two orders of magnitude greater than reported in vitro *K*_d_ values for binding of phenylboronic acid with monosaccharides^22^. These results suggest that a high effective molarity of diols is required to achieve substrate binding at micromolar concentrations of Rhobo6. At a Rhobo6 concentration of 5 µM, the time to reach 50% of the maximum signal was approximately 15 min, with more than 90% of signal achieved at 60 min, which led to our decision to use an incubation time of 1 h for biological samples.

Finally, the sensitivity of Rhobo6 labeling to photobleaching over repeated rounds of imaging was assessed. If, as expected, a reversible equilibrium existed between free and bound dye in a sample, the pool of excess free dye would replenish transiently bound photobleached molecules, resulting in a stable signal over time^20^. Indeed, using coated aggrecan as a substrate, no loss of fluorescence was observed over 9 h of acquisition at one frame per min (Extended Data Fig. 3b,c and Supplementary Video 1).

### Glycan-dependent labeling on cell surfaces

**Extended Data Fig. 4:**
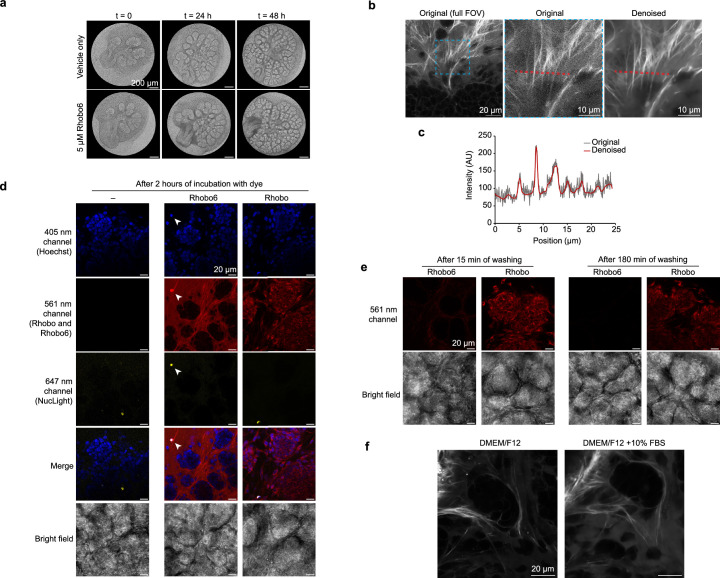
Rhobo6 toxicity and, reversibility in comparison to Rhobo, related to Fig. 3. **a**, Brightfield images of a representative pair of glands from Fig. 3b. **b**, Non-denoised (original) image of embryonic salivary gland labeled with Rhobo6, corresponding to the denoised version shown in Fig. 3c. Inset shows comparison between original and denoised dataset, confirming that artifacts were not introduced during the denoising process. Denoise was performed through Nikon NIS Elements AI Denoise (cf. Supplementary Table 1). Line plot of red region is reported in (**c**). **c**, Line plot of red outlined region from (**b**). Denoising increases signal to noise without altering biological features. **d**, Mouse embryonic salivary glands labeled with 5 µM Rhobo or Rhobo6, along with Hoechst (nuclear stain) and NucSpot650 (dead cell stain). Glands were incubated for 1 h with all probes and imaged. Rhobo6 labeling is confined in the extracellular space, but colocalizes with dead cells due to compromised membrane integrity (arrows, cf. Extended Data Fig. 3d). On the other hand, the cell permeable dye Rhobo labeling accumulates intracellularly in both live and dead cells. **e**, Glands from (**d**) were washed three times over the course of 3 h with DMEM/F12. Rhobo6 labeling was rapidly reversible, while Rhobo labeling was not diminished by washing over the course of the experiment. Rhobo images are contrast normalized across (**d**) and (**e**) and Rhobo6 images are independently normalized across (**d**) and (**e**). Notably, the cell permeable dye Rhobo is not able to label structures of the extracellular matrix in these glands, likely due to cytosolic sequestration. **f**, A salivary gland was incubated with 5 µM Rhobo6 in DMEM/F12, imaged, washed to remove Rhobo6 labeling, then incubated once again with Rhobo6 in DMEM/F12 containing 10% fetal bovine serum (FBS) and imaged. Source data

Next, Rhobo6 was applied at 5 µM in serum-free medium to an immortalized mammary epithelial cell line (MCF10A) in which the extent of cell-surface glycosylation could be predictably modulated through doxycycline-inducible expression of the heavily *O*-glycosylated transmembrane protein Mucin-1 lacking its carboxy-terminal cytosolic domain (MUC1∆CT)^35^. After 1 h of incubation with Rhobo6 at 37 °C, MUC1-dependent Rhobo6 labeling of cell surfaces was observed, and this signal was ablated when cells were pretreated with a mucin-selective protease (Fig. 2g)^36^. MUC1-dependent signal was reduced upon the addition of 200 mM sorbitol or serum-containing medium, the latter of which is expected to be rich in glycoconjugates (Extended Data Fig. 3d). Rhobo6 staining is not compatible with samples that are chemically fixed or that otherwise exhibit compromised cellular membranes, because the dye will internalize, resulting in intracellular fluorescence that drowns out cell-surface fluorescence signal (Extended Data Fig. 3d and Extended Data Fig. 4d).

Modulation of fluorescence lifetime upon changes of nitrogen atom substitution in rhodamine dyes has been reported^37^. Those results, combined with the observed increase in quantum yield upon diol binding in our study, suggested that free and bound Rhobo6 populations could exhibit measurable differences in their fluorescence lifetimes. Indeed, fluorescence lifetime imaging microscopy (FLIM) of MUC1-expressing cells enabled gating of populations, centered at 2 ns and 3.5 ns, corresponding to free and bound dye, respectively (Fig. 2h).

### Benchmarking Rhobo6 in excised tissues

**Fig. 3:**
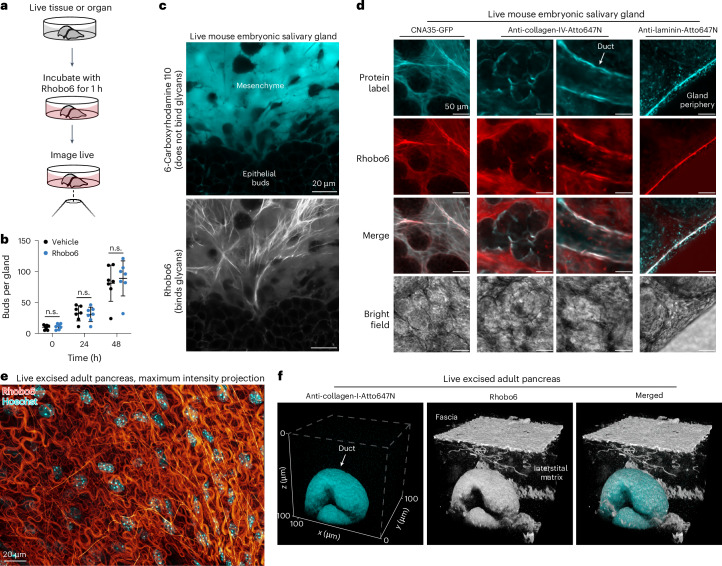
Labeling of excised tissues by bathing in Rhobo6-containing medium. **a**, Schematic of the labeling approach. Freshly dissected or cultured tissues were labeled with 5 µM Rhobo6 for 1 h, with sample-specific medium (Methods). **b**, Growth and morphogenesis of mouse embryonic salivary glands upon incubation with Rhobo6; *n* = 14 glands were split into two paired groups, with each pair corresponding to glands from a single embryo. The first group was incubated with 5 µM Rhobo6 in medium containing 0.5% DMSO, and the second group was incubated in medium containing 0.5% DMSO, as a vehicle control. Growth and morphogenesis were assessed by counting epithelial buds every 24 h for 2 days. Paired groups were compared using paired two-tailed *t*-tests; error bars represent s.d.; n.s., not significant. **c**, Mouse embryonic submandibular salivary gland (E14) was cultured ex vivo for 5 days, then labeled by bathing concurrently with Rhobo6 and 6-carboxyrhodamine 110. The latter dye differs from Rhobo6 only in that it does not contain the two *ortho*-aminomethylphenyl boronic acid groups, which are necessary for binding to extracellular glycans. Images were denoised (see Extended Data Figure 4b,c for comparison of raw and denoised salivary gland images; Supplementary Table 1 details the image-processing workflow for all datasets). **d**, Comparison of live Rhobo6 labeling to live labeling with protein-based affinity reagents against common ECM components, including fibrous collagen (CNA35), network-forming collagen (anti-collagen-IV), and laminins. Glands were incubated with purified CNA35-GFP or Atto647N-conjugated antibodies in solution along with Rhobo6, and were imaged using a confocal microscope. Contrast was not normalized. Images were denoised (Supplementary Table 1). **e**, Freshly dissected and exsanguinated mouse pancreatic tissue, labeled by bathing with Rhobo6 (red), to highlight ECM, and Hoechst (cyan), to highlight nuclei. The image shows a maximum intensity projection over a depth spanning 23 µm and showing fascia. Images were denoised (Supplementary Table 1). **f**, Two-color labeling of exsanguinated adult mouse pancreatic tissue labeled with both Rhobo6 (white) and an anti-collagen-I-Atto647N (cyan) antibody. Tissue was labeled by bathing for 1 h with both probes, and imaged with a confocal microscope. The image shows three-dimensional reconstruction of the 106 µm × 106 µm × 100 µm volume. Source data

To further benchmark Rhobo6, samples with complex, multicomponent extracellular matrices were labeled live through addition of dye to the native culture medium (Fig. 3a). Mouse submandibular salivary glands were isolated at embryonic day 13 or 14 (E13–E14) and cultured ex vivo. These glands continue to develop over the course of days in culture, undergoing budding and ductal morphogenesis^38^. To assess the biocompatibility of Rhobo6, growth and morphogenesis of paired salivary glands from seven embryos cultured with or without Rhobo6 over 48 h were assessed. Rhobo6 caused no difference in the overall morphology or the number of epithelial buds (Fig. 3b and Extended Data Fig. 4a), meaning that Rhobo6 is neither toxic nor perturbative to an embryonic organ explant.

Next, 6-carboxyrhodamine 110 and Rhobo6, each at 5 µM, were added to a salivary gland for 1 h at 37 °C. Live two-color imaging was enabled by the ~20 nm spectral separation of the two dyes (see above). The cell-impermeable fluorophore 6-carboxyrhodamine 110, which lacks boronic acids and cannot bind glycans, filled extracellular spaces similarly to dextran–fluorophore conjugates and charged small-molecule fluorophores that are routinely used for that purpose^32,39^ (Fig. 3c, top). By contrast, the boronic-acid-functionalized dye Rhobo6, which binds to glycans, revealed a network of fibrillar material surrounding epithelial buds and mesenchymal cells (Fig. 3c, bottom). Next, Rhobo6 was compared with the previously reported cell-permeable analog Rhobo. Rhobo could not label structures of the ECM, likely owing to depletion of the extracellular pool of dye following irreversible sequestration into epithelial and mesenchymal cells (Extended Data Fig. 4d,e). Finally, ECM labeling in salivary glands persisted in serum-containing medium, suggesting that the presence of serum is chiefly limiting for imaging cell-surface glycans (Extended Data Fig. 4f).

To explore the identities of the molecules underlying the observed Rhobo6 signal, glands were stained live with Rhobo6 and fluorescently labeled protein-based affinity reagents, followed by two-color imaging. In various fields of view, colocalization of Rhobo6 signal was observed with anti-collagen-IV antibody, anti-laminin antibody, and CNA35, a 39-kDa truncation of the collagen adhesion protein from *Staphylococcus* *aureus*, which binds various forms of fibrillar collagen (Fig. 3d)^40^.

As a second test system, adult mouse pancreatic tissue was excised and bathed in a buffer containing 5 µM Rhobo6 and 2 µg ml^–1^ Hoechst for 1 h. Confocal imaging of a 23-µm deep volume near to the tissue surface provided a view of the Rhobo6-stained fascia and embedded cellular nuclei, in a one-step, wash-free protocol (Fig. 3e). Because the preparation of excised pancreatic tissue was exsanguinated (Methods), live antibody labeling was possible. The tissue was bathed with a fluorophore-conjugated mouse antibody to collagen I alongside Rhobo6 for 1 h, then a 106 µm × 106 µm × 100 µm volume was acquired using a confocal microscope. The anti-collagen-I antibody labeled a pancreatic duct; Rhobo6 labeled the duct, surface fascia, and interstitial matrix (Fig. 3f), underscoring that Rhobo6 trades molecular specificity for a holistic view of ECM architecture.

**Extended Data Fig. 5:**
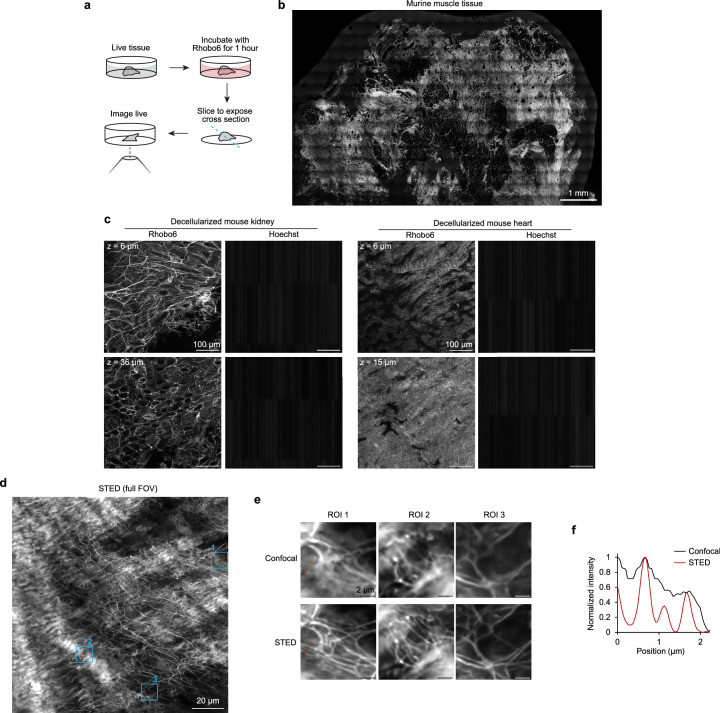
Rhobo6 diffusion in tissue, labeling of decellularized tissue, and compatibility with STED microscopy. **a**, Scheme for Rhobo6 diffusion experiment. A freshly excised tissue sample was incubated with Rhobo6 for 1 h, then removed from solution and sliced dry to expose a cross-section that was not in contact with the dye solution. The newly cut surface was placed face down on a dry coverslip and imaged. **b**, Mouse muscle tissue labeled as described in (**a**). **c**, Decellularized mouse tissues. Mouse kidney (*left*) and heart (*right*) slices were decellularized (cf. Methods), labeled with Rhobo6 and Hoechst for 1 h, then imaged. The absence of Hoechst labeling confirms successful decellularization. **d**, STED image of mouse pancreatic ECM obtained by imaging freshly excised tissue labeled with Rhobo6. Depletion was achieved with a 660 nm laser. **e**, Three regions of interest (ROIs) from (**d**), comparing confocal and STED imaging. Contrast is not normalized across imaging conditions and fields of view. Both confocal and STED images were denoised (cf. Supplementary Table 1). **f**, Intensity plot of red line from denoised images in (**e**) displaying the increased resolving power achieved by STED microscopy when compared to diffraction-limited confocal microscopy. Source data

To assess diffusion of Rhobo6 across larger areas of live tissue, whole mouse quadricep muscle was bathed in Rhobo6 for 1 h. ECM was labeled across a full ~6 mm cross section of the tissue that was not in contact with the dye (Methods), confirming rapid dye penetration and staining (Extended Data Fig. 5a,b). Next Rhobo6’s performance on decellularized tissues, which are often used in cases in which an ECM scaffold is required but cellular material is undesirable, was tested^41^. Mouse kidney and heart tissue were sectioned, decellularized (Methods), and incubated with Hoechst, which confirmed the absence of cellular material, and Rhobo6, which labeled ECM components successfully (Extended Data Fig. 5c). Finally, because Rhobo6 is a rhodamine-based dye, it is compatible with live super-resolution imaging of the ECM using stimulated emission depletion (STED) microscopy^42^, as was demonstrated in freshly excised mouse pancreatic tissue (Extended Data Fig. 5d–f).

### Rhobo6 applied to non-mammalian model organisms

Glycosylation is a feature of extracellular biomolecules across the kingdoms of life. To test Rhobo6’s performance in non-mammalian systems, we turned to *Drosophila melanogaster*, *Caenorhabditis* *elegans*, *Danio rerio*, and *Arabidopsis thaliana* model systems. In each case, two features were assessed: (1) cell impermeability and (2) the ability to label material in the ECM.

**Extended Data Fig. 6:**
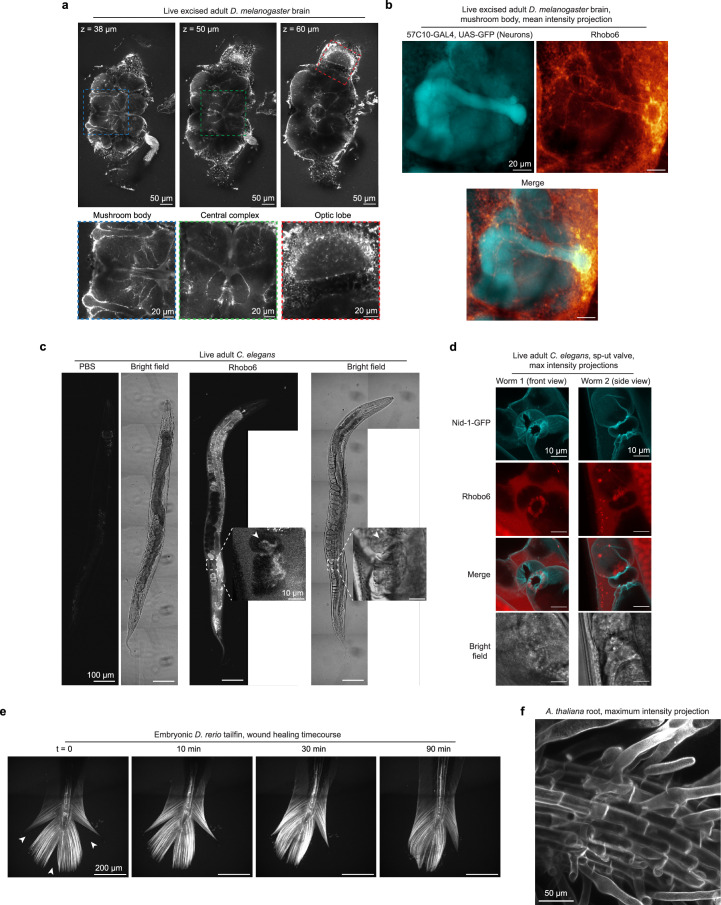
ECM labeling in non-mammalian organisms. **a**, Live, excised adult *Drosophila* brain was labeled by bathing with 5 µM Rhobo6 in saline. Imaging at different planes revealed labeling surrounding landmarks in the fly brain, including neuron tracts between the optic lobe and the central brain, the central complex, and the mushroom body (dashed boxes). Images were denoised (cf. Supplementary Table 1). **b**, Mean intensity projection of a confocal volume capturing the mushroom body in a *Drosophila* brain. The sample endogenously expressed GFP in neuronal cells and was labeled with Rhobo6 upon dissection as in (**a**) (cf. Methods). Rhobo6 labeling was excluded from cell interiors, as expected. Images were denoised (cf. Supplementary Table 1). **c**, Brightfield and confocal fluorescence images of a whole *C. elegans* injected with 10 pL of 100 µM Rhobo6 in PBS containing 1% DMSO. Contrast is normalized between PBS-injected and Rhobo6 injected animals. Inset is a crop and enlargement of the oviduct region, showing Rhobo6 labeling of that structure (arrows). **d**, Max intensity projections of confocal volumes taken at the oviduct of animals endogenously expressing Nidogen-1-mNeonGreen (Nid-1-GFP) to highlight oviduct surfaces, co-localized to Rhobo6 labeling. Rhobo6 signal is enriched at the sp-ut valve within the lumen of the oviduct. Rhobo6 does not label the Nid-1-rich oviduct basement membrane, which is not in contact with the lumen of gonad arm. **e**, Time course of wound healing in zebrafish larvae (8d.p.f.) incubated with 5 µM Rhobo6 in tank water. Tail nicks (arrows) were necessary for dye delivery. Structures of the tail ECM and notochord are labeled. **f**, Max intensity projection of an *Arabidopsis* root after labeling overnight with 5 µM Rhobo6 in pure water (cf. Methods). Root cell surfaces are labeled.

Adult *D. melanogaster* brains were excised into saline containing 5 µM Rhobo6, incubated for 1 h at room temperature, then imaged live. A pattern of labeling was observed that suggested targeting of structures that surround neuronal cells, such as those in the mushroom body, central complex, and optic lobe (Extended Data Fig. 6a). Two-color imaging using a fly line with neurons expressing cytosolically targeted green fluorescent protein (GFP) confirmed that Rhobo6 labeling was excluded from cell interiors (Extended Data Fig. 6b). Next, adult *C. elegans* worms were injected with 10 pL of 100 µM Rhobo6 in each proximal arm of the gonad. Structures including yolk, eggshells, and the vulva were labeled (Extended Data Fig. 6c)^43^. A labeling pattern that appeared to be in the oviduct was also observed. Two-color imaging with Rhobo6 in a worm line expressing endogenously tagged Nidogen-1-GFP^44^ revealed that the signal was concentrated at the spermathecal–uterine valve within the lumen of the cell, not in its cytosol (Extended Data Fig. 6d). In larval zebrafish (8 days post fertilization (d.p.f.)), Rhobo6 was added at 5 µM to tank water and delivered through incisions to the tail. Rhobo6 visualized structural ECM components in the tail and notochord of the fish during a time-lapse of wound healing (Extended Data Fig. 6e and Supplementary Video 2). Finally, *A. thaliana* seedlings were grown from seed on agar (Methods). Seedlings were watered with 5 µM Rhobo6, incubated overnight, then imaged. Rhobo6 signal localized to root cell surfaces, consistent with previously observed distributions of metabolically incorporated azido-monosaccharides (Extended Data Fig. 6f)^45^. Taken together, these data confirm that Rhobo6 is compatible with a wide array of biological samples using a wash-free labeling protocol.

### Tissue distribution of Rhobo6 upon injection in mice

**Fig. 4:**
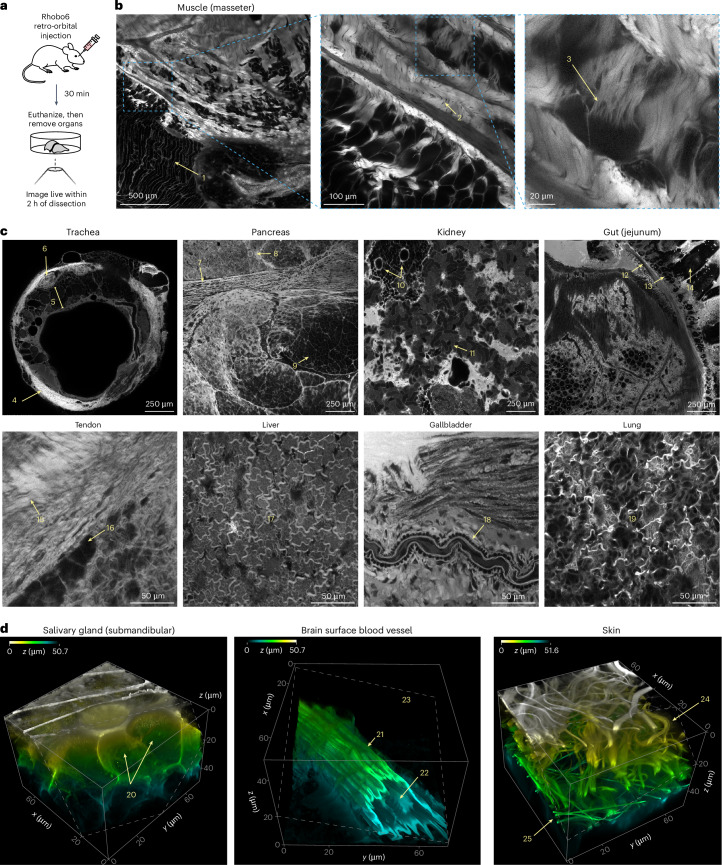
Rhobo6 is distributed across mouse organs and labels the ECM upon retro-orbital injection. **a**, Schematic of the labeling approach. Anesthetized mice were injected retro-orbitally with 100 µl of 1 mM Rhobo6 in PBS containing 10% DMSO, which corresponds to 100 nmol of Rhobo6, or 3.5 mg kg^–1^ body weight for a 20 g mouse. Mice were allowed to recover for 30 min on a warming pad, and were then euthanized by cervical dislocation. Live tissues were collected, placed on a glass-bottom dish, and imaged within 2 h of dissection. **b**, 2P image of a 2 mm by 2 mm area of muscle tissue (masseter). Insets show sequential crops of the original image, highlighting ECM features made visible by Rhobo6 labeling. For annotations of numbered landmarks, see **c**. **c**, Individual fields of view cropped from 2 mm by 2 mm 2P images of the indicated tissues. Numbers in yellow correspond to features consistent with histological annotations^56,57^. Muscle (in **b**): (1) skeletal muscle fibers, (2) collagen-rich fascia, and (3) basal lamina surrounding myofibrils. Trachea: (4) tracheal cartilage ring, (5) submucosal layer with basement membrane, and (6) a tracheal gland encased in ECM. Pancreas: (7) longitudinal section of a duct, (8) the cross section of a duct, and (9) acinar tissue. Kidney: (10) collecting tubule and (11) convoluted tubules. Jejunum: (12) muscularis mucosa, (13) crypts, and (14) villi. Tendon: (15) fascia and (16) fibroblasts. Liver: (17) the entire field of view shows the fascia layer superficial to the hepatocyte layer. Gallbladder: (18) longitudinal section of an arteriole. Lung: (19) the entire field of view shows alveolar tissue encased in ECM. All tissues, including images in **b** and **d**, were acquired on the same day from four mice of the same strain and age (Methods). Contrast was not normalized across samples. **d**, Three-dimensional reconstructions of three tissues, from 2P microscopy volumes. Histological annotations are numbered in yellow. Salivary gland: (20) epithelial bud cell interiors from which Rhobo6 is excluded. Brain: (21) a blood vessel on the brain surface, (22) red blood cells that are excluded from Rhobo6 labeling within the vessel and (23) brain tissue that is not labeled by Rhobo6, therefore appearing dark. Skin: (24) collagen fibers and (25) elastin fibers. Contrast and the depth-coded lookup table were not normalized across samples. Images in **d** were denoised (Supplementary Table 1).

Given the absence of toxicity observed in developing salivary glands exposed to Rhobo6 (Fig. 3b), we next investigated whether Rhobo6 could be administered to mice through injection (Fig. 4a). Retro-orbital injection of 100 nmol (~3.5 mg kg^–1^ body weight) Rhobo6 did not result in apparent toxicity in 8- to 12-week-old female C57BL6/J mice. To assess the distribution of the dye, the animals were euthanized 30 min after injection, and excised organs were placed on glass coverslips for imaging (Methods). A panel of 12 live tissues was imaged in this fashion using 2P microscopy; 2 mm × 2 mm areas or approximately 70 µm × 70 µm × 50 µm volumes were acquired. Labeling of structures in the ECM was observed in all tissues except for brain, in which dye is likely excluded by the blood–brain barrier (Fig. 4b–d and Supplementary Videos 3 and 4; numbered arrows indicate tissue landmarks described in the caption for Fig. 4c,d). These images and volumes underscore the heterogeneity of ECM structures across tissues of the mouse and the broad distribution of Rhobo6 across organs, including relatively low blood flow areas such as tendon. Additionally, the presence of blood serum in these tissues did not interfere with Rhobo6 contrast, likely owing to the higher effective molarity of available diols in tissue ECM relative to cultured cell surfaces.

**Extended Data Fig. 7:**
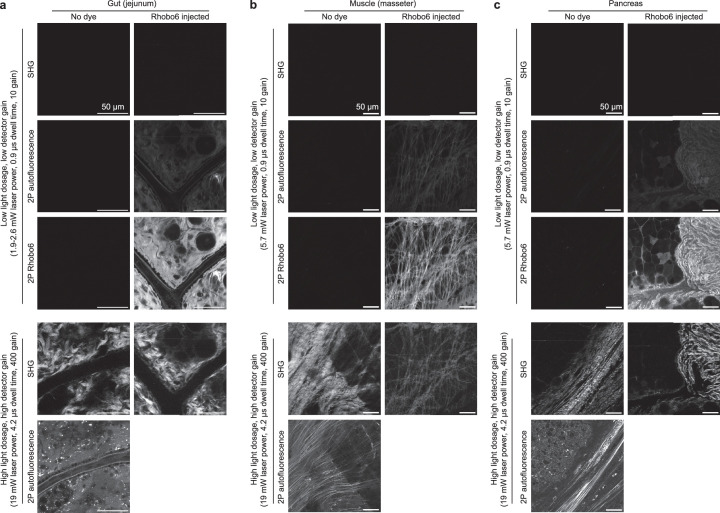
Comparison between 2-photon Rhobo6 imaging and label-free ECM imaging techniques. **a-c**, Gut (jejunum), muscle (masseter), and pancreas from both a control mouse and a mouse retro-orbitally injected with 100 µl of 1 mM Rhobo6 in PBS containing 10% DMSO, which corresponds to 100 nmol of Rhobo6, or 3.5 mg/kg for a 20 g mouse. Rhobo6-injected animals were imaged using second harmonic generation microscopy (SHG), two photon excitation autofluorescence (2 P autofluorescence, also known as TPEF) and two photon excitation fluorescence (2 P Rhobo6). Fields of view were imaged with a low light dosage highlighting the efficiency of 2 P Rhobo6 compared to SHG and TPEF, followed by a high light dosage for SHG and TPEF, in order to obtain contrast with those methods (cf. Supplementary Table 1). Contrast was normalized across all low dosage images within each tissue type. High dosage SHG images are normalized to each other and high dosage TPEF images are not normalized to any other image. Reported laser power is the average measured power at the sample plane for 120 fs pulses at 80 MHz repetition rate.

For a subset of tissues, head-to-head comparisons were performed with second-harmonic-generation microscopy and 2P autofluorescence imaging, which are often used to image collagen and elastin, respectively^6^. Rhobo6 enabled visualization of both collagen and elastin structures simultaneously using lower doses of light (Extended Data Fig. 7).

### Imaging of tumor-driven ECM remodeling

**Fig. 5:**
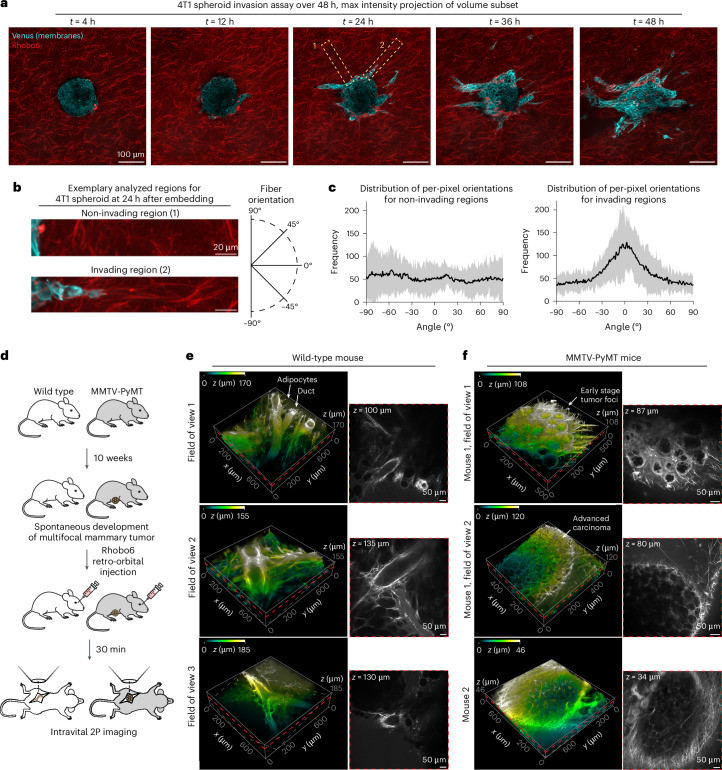
Imaging of ECM in matrix-embedded breast cancer spheroids and in a mouse model of breast cancer. **a**, Spheroid invasion assay time course. 4T1 spheroids expressing membrane-tethered Venus were embedded in an ECM scaffold (80% collagen type I, 20% Matrigel), then bathed in serum-containing medium with 5 µM Rhobo6. Volumes of 553 µm × 553 µm × 162 µm were acquired once per hour over 2 days using 2P microscopy. Maximum intensity projections over 12 µm are shown. Yellow boxes represent examples of non-invading (1) and invading (2) regions shown in **b**. **b**, Left, example non-invading and invading spheroid regions used for fiber-orientation analysis, cropped from **a** (Methods). Right, frame of reference for quantification, in which fibers of 0° are parallel relative to the long axis of the ROI. **c**, Distribution of per-pixel orientations from −90° to 90° in non-invading (left) and invading (right) ROIs, plotted as an average of *n* = 3 regions for each of *n* = 3 spheroids. Orientation was evaluated for every pixel on the basis of the structure tensor using OrientationJ^58^. Shaded bands indicate the s.d. **d**, Schematic of the experimental timeline, along with the intravital imaging strategy for wild-type and mammary tumor-bearing MMTV-PyMT mice. **e**, Rhobo6 imaging with three fields of view from the same mammary gland marking the ECM surrounding normal ductal architecture. Left, volume rendering (color-coding for depth was applied). Arrows indicate adipocytes and epithelial ducts. Right, a single confocal slice from the adjacent volume (red dashed plane, with *z* height indicated) illustrating Rhobo6 labeling. Contrast is not normalized. Images were denoised (Supplementary Table 1). **f**, The same as in **e**, for two MMTV-PyMT mice. Two fields of view are presented for mouse 1 and one field of view is shown for mouse 2. Arrows indicate early-stage and late-stage carcinomas. Contrast is not normalized. Images were denoised (Supplementary Table 1). Source data

**Extended Data Fig. 8:**
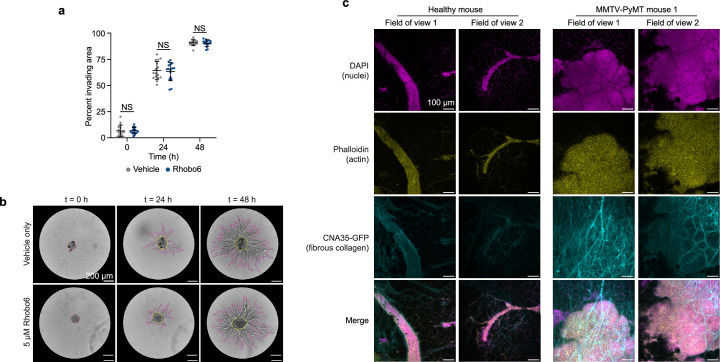
Rhobo6 influence on invasive potential of 4T1 spheroids and immunostaining of wild-type and tumor-bearing mouse mammary glands, related Fig. 5. **a**, Spheroids were incubated with media containing 0.5% DMSO (vehicle) or with media containing 5 µM Rhobo6 and 0.5% DMSO (N = 16 spheroids per condition). Spheroids were imaged with a widefield microscope at t = 0 after embedding and incubation, then every 24 h for 2 days, and percent invading area was calculated (cf. Methods). Statistical analysis was performed via two-way ANOVA and Sidak’s multiple comparison test, with the assumption of a single pooled variance; NS = not significant. **b**, Brightfield images of representative organoids from (a). Traced regions corresponding to the core spheroid area (yellow) and traced regions corresponding to the entire spheroid, including all invading protrusions (magenta), were used to calculate percent invading area (cf. Methods). **c**, Immunofluorescence of fixed and wholemounted mammary glands using phalloidin (yellow) and CNA35-GFP (cyan) to mark filamentous actin and fibrillar collagen, respectively, and DAPI to stain cell nuclei (magenta). Two fields of view are shown for each of the mammary glands presented in Fig. 5, resected from the wild-type mouse (left) and MMTV-PyMT mouse 1. Source data

Malignancy in mammary tissue is accompanied by profound changes to the ECM, such as the accumulation and remodeling of fibrillar collagen into dense, linear and stiffened fibers^46–48^. To assess these ECM alterations over time, Rhobo6 was used in a spheroid invasion assay in the mouse breast cancer cell line 4T1 expressing a membrane-tethered GFP (Venus). Tumor spheroids were embedded in a mixture of collagen I and Matrigel, and were then incubated in serum-containing medium. The presence of Rhobo6 in culture medium did not affect spheroid invasive potential, providing further evidence for Rhobo6’s biocompatibility (Extended Data Fig. 8a,b). Next, two-color, 2P volumes of *n* = 3 spheroids were acquired at 1-h intervals over 2 days under incubation, without exchange of medium (Fig. 5a and Supplementary Video 5). Quantification of ECM fiber orientations at 24 h after embedding revealed that fibers of invading regions oriented parallel to protruding cells, whereas fibers of non-invading regions did not exhibit a directional preference (Fig. 5b,c). These results are consistent with prior observations, which functionally link the presence of fibers oriented parallel to cancer-cell protrusions with tumor invasion^49^.

Next, intravital imaging was performed with Rhobo6 to compare ECM phenotypes at different stages of cancer progression. Mouse mammary tumor virus (MMTV)-driven polyoma middle T oncoprotein (PyMT) mice, a well-characterized genetically engineered mouse model of breast cancer, were used. PyMT rapidly induces spontaneous multifocal tumors in mice in a manner that is comparable to the stages of progression observed in human disease^50^. Mice were imaged at 10 weeks of age, when approximately 50% of MMTV-PyMT mammary glands contained advanced late carcinoma along with a mixture of adenoma/mammary intraepithelial neoplasia (MIN) and early carcinoma^50^ (Fig. 5d). In live wild-type mice, Rhobo6 labeled the ECM surrounding ductal epithelium and fibrillar structures between stromal adipocytes (Fig. 5e). This contrasts with Rhobo6 imaging in MMTV-PyMT glands, which demonstrated substantial alterations to ECM architecture (Fig. 5f). At an adenoma/MIN or early stage of carcinoma (Fig. 5f, top), there was a thick basement membrane around malignant foci and an increased presence of ECM between individual foci. In more advanced carcinoma, a basket-like network of fibrillar ECM surrounding tumor nodules was observed, with many fibers oriented more perpendicularly rather than tangentially to the tumor margins, paralleling observations in the spheroid model.

Following intravital imaging, whole mammary glands were resected and fixed for histological analysis. Immunofluorescence labeling of actin (phalloidin) and cell nuclei (DAPI) was performed to visualize cellular architecture and localize malignant regions. In addition, CNA35 was included for comparison with Rhobo6-derived images. Differences in tissue architecture between mammary tumors and healthy ductal epithelium were apparent from the actin and nuclear labeling (Extended Data Fig. 8c). Moreover, CNA35-mediated labeling demonstrated collagenous structures that corresponded well with the intravital imaging, with substantial infiltration of ECM and stroma between tumor foci and thick fibers oriented at increasing angles from the tumor margin (Extended Data Fig. 8c). These studies confirm the ability of Rhobo6 to efficiently label ECM in vivo and effectively distinguish tumor-associated ECM from healthy ECM structures in an intravital imaging setting.

## Discussion

Our aim was to develop a method that enables one-step, wash-free visualization of ECM architecture in a wide variety of biological samples. To achieve that goal, we developed Rhobo6, a cell-impermeable small-molecule fluorophore that turns on and red shifts upon reversible binding to glycans, a nearly universal feature of ECM biomolecules. Rhobo6 has a number of characteristics that warrant discussion and will inform use cases.

First, a key enabling feature of Rhobo6 is its cell impermeability, which prevents irreversible intracellular accumulation and subsequent depletion of extracellular dye (Extended Data Fig. 4d,e). As such, Rhobo6 is incompatible with cellular samples that have been chemically fixed or in which plasma membranes have been otherwise compromised, unless the sample is decellularized (Extended Data Figs. 3d and 5c). Rhobo6’s cell impermeability also implies that delivery into whole organisms is likely to require injection or disruption of outer barriers (for example, zebrafish tail incision, Extended Data Fig. 6e).

Second, the affinity of boronic acid groups for single monosaccharides is low, with dissociation constants expected to be in the range of tens of millimolar^22^. Therefore, the high, local effective molarity of glycans in a biological sample likely drives the observed pattern of labeling (the effective *K*_d_ is estimated to be 53 µM on a purified substrate; Fig. 2e,f). When Rhobo6 is applied at low micromolar concentrations to a sample, an equilibrium exists between free and bound dye, with an excess of free dye available to replenish bound molecules. Such reversible, low-affinity binding likely enables Rhobo6 to be minimally perturbative to native ECM structures in tissues, as demonstrated in the developing embryonic salivary gland and spheroid invasion assay (Fig. 3b and Extended Data Figs. 4a and 8a,b). This equilibrium also prevents photobleaching (Extended Data Fig. 3b,c and Supplementary Video 1), an advantage for studying ECM dynamics, which often occur over long timescales^1,2^ (Fig. 5a). Moreover, Rhobo6 labeling is reversible upon buffer exchange (Extended Data Fig. 4e), meaning that repeated rounds of washing and labeling should enable spectral multiplexing and imaging at multiple time points in a single sample^20^.

Third, Rhobo6 is designed to selectively bind extracellular glycans, but it does not exhibit specificity for any one glycan or ECM component (see glycan array, Extended Data Fig. 2). Rather, Rhobo6 broadly labels glycoconjugates of the ECM with a range of intensities that depend on the abundance of the underlying biomolecules, the effective affinity of Rhobo6 to each biomolecule, and environmental factors (for example, the presence of serum, pH). For example, Rhobo6 seems to label the glycocalyx of cultured cells dimly (see MCF10A cells lacking MUC1∆CT expression, Fig. 2g and Extended Data Fig. 3d), suggesting that methods such as metabolic incorporation and chemoenzymatic labeling^16^ will remain preferable to Rhobo6 for labeling the glycocalyx. Generally, in cases where hypotheses related to purely structural or mechanical properties of the ECM (for example fiber orientation, Fig. 5c) are being tested, Rhobo6 imaging will provide quantitative measurements. In cases where hypotheses require molecular information about ECM composition, Rhobo6 is best used as a starting point to be followed by tools that are more specific to the target. In addition, image acquisition parameters and image display settings might need to be adjusted to highlight different ECM components.

Overall, Rhobo6 provides a holistic view of ECM architecture at the cost of molecular specificity. By analogy, live-cell nuclear stains, such as Hoechst, take advantage of the fact that the nucleus is rich in a class of fundamental biopolymer (DNA) that has a unique motif (a minor groove). Although the specificity of Hoechst to DNA is complicated by preferences for base-pair sequences and minor-groove accessibility, in most sample types, there is a sufficient quantity of substrate sites available for the dye to be used to visualize the distribution of nuclei in tissues^21^. Rhobo6, meanwhile, takes advantage of the fact that the ECM is rich in a different fundamental biopolymer (glycans) that also has a unique motif (1,2- and 1,3-diols). We envision that Rhobo6 will find use as a straightforward and reliable stain for visualizing the distribution of ECM in tissues, including those that are not amenable to genetic manipulation and/or ex vivo culture.

Looking ahead, opportunities exist for further development of phenylboronic-acid-modified fluorophores as ECM labels, including endowing glycan selectivity^25^, enhancing oxidative stability^22^, and tuning spectral properties^28,51^. Molecular design efforts would be aided by characterization of the mechanism underlying both the fluorogenicity and spectral shift of Rhobo6 in the presence of diols. Upon diol binding, the majority of reported *ortho*-aminomethylphenyl-boronic-acid-functionalized dyes exhibit fluorescence turn on, but do not red shift^31^. Rhobo6 differs from these molecules in that the *ortho*-aminomethyl group is directly attached through its nitrogen atom to the conjugated system of the fluorophore. Notably, a molecule synthesized by Shinkai in 1995, composed of an *ortho*-aminomethyl group attached in a similar fashion to the conjugated system of a coumarin, also showed a spectral red shift upon sugar binding^52^.

There are opportunities to apply Rhobo6 using fluorescence imaging and data-analysis modalities aside from those presented here. A low concentration of Rhobo6 applied to relatively immobile samples might allow points accumulation for imaging in nanoscale topography (PAINT) microscopy, which provides nanometer-precision single-molecule localizations^53,54^. Generation of a large ground-truth dataset of known labels colocalized with Rhobo6 could open the door to machine-vision annotation of ECM components on the basis of properties such as persistence length and cellular context, providing a degree of molecular information in single-color Rhobo6 images^55^.

Finally, our intravital imaging results suggest that Rhobo6 or future analogs could find utility as diagnostic tools for biopsy samples, in diagnostic imaging, or in fluorescence-guided surgery. To explore that possibility, dye pharmacokinetics will need to be characterized, and fluorescence contrast in clinical settings will need to be assessed.

## Methods

### Microscopy methods

Supplementary Table 1 tabulates microscopy platforms, imaging parameters, and data-processing steps for all datasets. Unless noted otherwise, image processing was performed in Fiji/ImageJ (National Institutes of Health).

#### Considerations for multiplexing

Rhobo6, particularly in the unbound state, can be excited by a 488-nm laser line to some degree (Extended Data Fig. 1e–h). As a result, multiplexing with green fluorophores, for example GFP, requires attention to emission filters to minimize fluorescence bleed-through. In particular, we typically used a cut-off wavelength of 525 nm for the green emission filter, while keeping the Rhobo6 emission filter above 575 nm (Supplementary Table 1). For multiplexing with far red probes, such as Atto647N, we set the upper cut-off for the Rhobo6 emission filter to 630 nm.

### Organic synthesis and chemical characterization

#### General considerations

All chemicals, in reagent grade or higher, were obtained from commercial suppliers and used as received. Reactions were conducted in 2- to 5-ml Biotage microwave vials sealed with Biotage microwave-proof caps and heated in a Biotage Initiator+ microwave synthesizer. Reactions were monitored by liquid chromatography–mass spectrometry (LC–MS) (Shimadzu SIL-20ACHT with Phenomenex Kinetex 2.1 × 30 mm 2.6 µm C18 column; 2–10 µl injection; 5–98% acetonitrile/H_2_O, linear gradient, with constant 0.1% vol/vol HCO_2_H additive; 6 min run; 0.5 ml min^–1^ flow; ESI; positive-ion mode). Reaction products were purified by preparative HPLC (Shimadzu SPD-M20A with Phenomenex Gemini 30 × 150 mm 5 µm NX-C18 column). Analytical high-performance LC (HPLC) analysis was performed using an Agilent Technologies 1200 Series with Agilent Eclipse 4.6 × 150 mm, 5 μm XDB-C18 column under the indicated conditions. High-resolution MS (HRMS) was performed by the High Resolution Mass Spectrometry Facility at the University of Iowa. Nuclear magnetic resonance (NMR) spectra were recorded on a Bruker Avance II 400 MHz spectrometer. Chemical shifts are reported in parts per million (ppm) relative to residual solvent peaks. ^1^H NMR data are presented as follows: chemical shift (*δ* ppm), multiplicity (s, singlet; d, doublet; dd, doublet of doublets; m, multiplet), coupling constant in Hertz (Hz), integration.

(E)-2-(6-((2-boronobenzyl)amino)-3-((2-boronobenzyl)iminio)-3H-xanthen-9-yl)benzoate (Rhobo) was synthesized as previously reported^59^.

#### Synthesis of (E)-2-(6-((2-boronobenzyl)amino)-3-((2-boronobenzyl)iminio)-3H-xanthen-9-yl)-4-carboxybenzoate (Rhobo6)

6-Carboxyrhodamine 110 (HCl salt, 51 mg, 0.124 mmol, 1 equivalent (eq)), 2-formylphenylboronic acid (100 mg, 0.667 mmol, 5.4 eq), sodium triacetoxyborohydride (90 mg, 0.425 mmol, 3.4 eq), and anhydrous DMF (1.7 ml) were added to a microwave vial containing a magnetic stir bar. The mixture was homogenized by ultrasonication at room temperature for 1 min, and concentrated acetic acid (50 μl, 0.874 mmol, 7.0 eq) was added. The vial was sealed with a microwave-proof cap and stirred at room temperature for 1 min. The reaction was then run at 130 °C for 60 min in the microwave synthesizer. After the vial was cooled to room temperature, the cap was removed, and ~20 ml of methanol was added to dilute the reaction mixture. Purification by preparative HPLC (25–50% acetonitrile/H_2_O, linear gradient, with constant 0.1% vol/vol trifluoroacetic acid (TFA) additive) yielded red Rhobo6 solid (TFA salt, 33.2 mg, 35%). ^1^H NMR (400 MHz, CD_3_OD): *δ*, 8.37–8.33 (m, 2H), 7.95−7.92 (m, 1H), 7.45–7.29 (m, 8H), 7.08 (d, *J* = 9.2 Hz, 2H), 6.89 (dd, *J* = 9.2, 2.2 Hz, 2H), 6.80 (d, *J* = 2.2 Hz, 2H), 4.61 (s, 4H). Analytical HPLC: retention time (*t*_R_) = 10.4 min, 95.4% purity (10–95% acetonitrile/H_2_O, linear gradient, with constant 0.1% vol/vol TFA additive; 20 min run; 1 ml min^–1^ flow; ESI; positive-ion mode; detection at 254 nm); HRMS (ESI) calculated for C_35_H_28_B_2_N_2_O_9_ [M+H]^+^ 643.2054, found 643.2065. For ^1^H NMR and analytical LC–MS spectra, see Supplementary Figure 1a,b. For stability over time, see Supplementary Figure 1c.

#### Dye storage

Freshly prepared Rhobo6 solid was dissolved in anhydrous DMSO at 10 mM, and subsequently distributed into 10-µl aliquots in screw-top vials, which were stored at −80 °C. Unless noted, aliquots were thawed at room temperature, diluted with anhydrous DMSO to 1 mM, then frozen once again at −80 °C. Then, 1 mM DMSO aliquots were diluted 1:200 into sample buffer to yield working concentrations of 5 µM, and were freeze–thawed five or fewer times before being discarded. For mouse injections, a 10-µl aliquot of 10 mM Rhobo6 in DMSO was diluted to 100 µl with sterile PBS.

#### Stability of Rhobo6 assessed by HPLC

An aliquot of Rhobo6 (10 µl of 10 mM in DMSO) was diluted with DMSO (40 µl) and PBS (50 µl) to a final concentration of 1 mM and then stored at room temperature in darkness. Its purity was assessed at 0, 1, 2, 4, 8, 24, 48, and 72 h by an analytical HPLC (Phenomenex Gemini 4.6 × 250 mm 5 µm NX-C18 column; 5 µl injection; 5–95% acetonitrile/H_2_O, linear gradient, with constant 0.1% vol/vol TFA additive; 25-min run; 1 ml min^–1^ flow; detection at 254 nm).

#### Monosaccharides and monosaccharide analogs

Solutions of d-glucose (Millipore Sigma, G7021), d-galactose (Fisher Scientific, BP656-500), d-mannose (Millipore Sigma, M8574), d-fructose (Millipore Sigma, F0127), and sorbitol (Millipore Sigma, PHR1006) were prepared at 400 mM in 0.5 ml PBS (Corning, MT21040CV), with pH adjusted to 7.3–7.4 using a pH meter (Mettler Toledo S470 coupled with Ultra-Micro-ISM pH probe). Three replicate Rhobo6 solutions were prepared at 10 µM in PBS, starting from three separate dye aliquots. The sugar substrate solution and Rhobo6 solutions were mixed 1:1 to yield three replicate mixtures at a final concentration of 5 µM Rhobo6 and 200 mM substrate. Dilution was performed directly in a 96-well black plate (Greiner Bio-One, 655900), followed by 1 h of incubation. Fluorescence emission was then measured using a Tecan Spark plate reader, with 555 nm excitation and an emission range of 570–630 nm in steps of 2 nm, and an integration time of 40 µs. As controls, both PBS (buffer only) and each of the dye solutions (dye only) were also acquired. Spectra were then analyzed by subtracting background counts (estimated by the buffer-only signal at each wavelength) and averaging the maximum signal for each spectrum across the three measurements.

### Photophysical characterization

Rhobo6 solutions were prepared by diluting 1 mM dye stock in either PBS or PBS solution containing 2M sorbitol (Millipore Sigma, S1876). Absorbance measurements were performed using a UV-Vis spectrometer (Cary 100, Agilent Technologies) and 5 µM dye. Extinction coefficients were calculated at peak absorbance in both conditions. Fluorescence emission spectra were measured using a spectrofluorometer (Cary Eclipse, Varian) with excitation at 490 nm. Quantum yield measurements were conducted using an integration sphere spectrometer (Quantaurus), averaging values measured between 475 and 535 nm at 5-nm increments. To measure contrast at different excitation wavelengths, we acquired emission spectra for both bound and unbound solutions with excitation at 490 nm and 561 nm. The reported contrast Δ*F*/*F*_561ex/575em_, that is the fluorescence contrast at an excitation wavelength of 561 nm and emission wavelength of 575 nm, was calculated as relative signal change at 575-nm emission, calculated from the 561-nm excitation dataset. To renormalize spectra to a given excitation wavelength, both emission spectra were normalized to a wavelength-specific excitation coefficient. This coefficient was estimated as:

$${{{E}}}_{{{\lambda }}{\rm{ex}}} \sim \varPhi (1-{10}^{-{{{A}}}_{{{\lambda }}{\rm{ex}}}})$$

*E*_*λ*ex_ denotes the estimated excitation at a given excitation wavelength (*λ*), *Φ* denotes the measured quantum yield, and *A*_*λ*ex_ denotes the measured absorbance at *λ*.

#### Two-photon excitation

Two-photon excitation spectral measurements were performed following established methods^60^. Dye solutions at 1 µM concentration were prepared in 100 mM phosphate buffer (pH 7.4) or the same buffer containing 1 M galactose. Spectral measurements were performed using an inverted microscope (IX81, Olympus) equipped with a ×60, 1.2-NA water immersion objective (Olympus). Dye samples were excited using pulses from an 80 MHz Ti-Sapphire laser (Chameleon Ultra II, Coherent) in the range of 710–1,080 nm, and with an OPO (Chameleon Compact OPO, Coherent) in the range of 1,000–1,500 nm. Fluorescence collected by the objective was filtered through a dichroic (675DCSPXR, Omega and fF825-SDi01, Semrock) and an emission (FF01-539/278-25 and FF01-709/167-25, Semrock) filter, before detection by a fiber-coupled Avalanche Photodiode (APD) (SPCM_AQRH-14, Perkin Elmer). Two-photon excitation spectra were obtained from 1 µM dye samples at 1 mW of laser power across the spectral range of 710 nm to 1,080 nm using Ti:Sapphire, and at 2 mW of laser power for spectral range of 1,000–1,500 nm using OPO. The excitation spectra have been normalized for the laser power and corrected for (1) the transmission curves of the dichroic and emission filters and (2) the quantum efficiency of the detector as a function of wavelength. We acquired and reported a 2P excitation spectrum for Rhodamine B, which was measured in the same manner and is also reported in the literature^61^, as a control. All spectra are averages of *n* = 2 measures.

### Rhobo6 binding to sorbitol at pH 6–8

Solutions of 100 mM phosphate buffer containing 0, 2, 20, 200, and 2,000 mM sorbitol were prepared at pH 6, 7, and 8. Solutions of 10 µM Rhobo6 in 100 mM phosphate buffer were prepared at pH 6, 7, and 8. The sorbitol and Rhobo6 solutions were mixed in a glass-bottom 96-well black plate (Corning, 354640) at a 1:1 ratio (total volume, 100 µl) for each pH. A negative control with 100 µl of 100 mM buffer at the target pH without dye was also included. After incubating the plate in the dark at room temperature for 1 h, the emission spectrum of each solution was acquired on a Tecan Spark X3 plate reader, and the collected data were subsequently normalized and plotted using MATLAB R2022a (MathWorks).

### Glycan array

To test the specificity of glycan binding across various glycoconjugates, we used a glycan array (RayBiotech, GA-Glycan-100-1), which is manufactured as a glass slide divided into four wells each containing one array; each array consists of 100 glycans printed in 4 replicate spots, along with 2 sets of 4 negative control spots. The glass slide was equilibrated from storage temperature (−20 °C) to room temperature for 90 min, then wells were rehydrated through incubation in PBS for 60 min. Subsequently, PBS buffer was replaced with 5 µM Rhobo6 solution in PBS. Three arrays were incubated for at least 90 min before being imaged sequentially with a confocal microscope. Fluorescence signal and local background for each glycan were quantified using MATLAB R2022a (MathWorks), which was followed by background-corrected signal normalization within each array to account for changes in background intensity conditions. Data were visualized and analyzed for statistical significance using Prism v.10.3.1 (GraphPad); statistical significance was determined through unpaired two-tailed *t*-test with Welch’s correction, relative to the negative control group.

### Purified components of the extracellular matrix

#### Coatings

Human fibronectin (Corning, 354008) was resuspended in water at 1 mg ml^–1^, following the manufacturer’s instructions, and was then used or aliquoted and frozen at −20 °C. Laminin (Thermo Fisher Scientific, 23017015) was supplied at 0.5–2 mg ml^–1^ in 50 mM Tris-HCL (pH 7.4) and 0.15 M NaCl, and was aliquoted and stored at −20 °C. Laminin isoforms 111LN and 521LN (Biolamina, LN111-02 and LN521-02) were supplied at a concentration of 0.1 mg ml^–1^, aliquoted, and stored at −20 °C. Collagen type IV (Corning, 354233) was supplied at 1.1 mg ml^–1^, aliquoted, and stored at −20 °C. Lyophilized aggrecan (Millipore Sigma, A1960-1MG) was resuspended at 2 mg ml^–1^ in PBS, aliquoted, and stored at −20 °C. After thawing an aliquot of each solution at 4 °C, 10 µl of each substrate was deposited onto an untreated glass-bottom well (Ibidi, 80807) and dried at 37 °C for 4 h before use.

#### Collagen gels

Collagen I gels at 1.5 mg ml^–1^ were prepared starting from collagen type I (Ibidi, 50201), thawed at 4 °C, and diluted in 17.5 mM acetic acid to 4 mg ml^–1^. A solution was then prepared with 6.67% 10× DMEM (Millipore Sigma, D2429-100ML), 0.67% NaOH 1 M solution in water, 18.5% distilled water, 3.33% of NaHCO_3_ 89 mM solution in water, 33.33% of 1× DMEM (Thermo Fisher Scientific, 21041025), and 37.5% of collagen I solution at 4 mg ml^–1^. The solution was quickly distributed in a 20-µl droplet in an untreated glass-bottom well (Ibidi, 80807) and incubated at 37 °C, 95% humidity, and 5% CO_2_ for at least 1 h before use.

#### Hyaluronan gels

Ninety microliters of 0.25 M NaOH aqueous solution was added to high-molecular-weight hyaluronan (1,500 kDa, 10 mg, R&D Systems) in a 1.5-ml Eppendorf microcentrifuge tube. The mixture was centrifuged at 3,000*g* and vortexed for 2 min until the hyaluronan was dissolved. 1,4-Butanediol diglycidyl ether (BDDE, 1 μl) was diluted with 0.25 M NaOH aqueous solution (10 μl) and then added to the hyaluronan solution. The mixture was centrifuged at 3,000*g* and vortexed for 30 s, and then centrifuged at 20,000*g* for 5 min to get rid of bubbles. Two microliters of solution were pipetted onto each well of an eight-well plate. The well plate was surrounded with water to prevent the hyaluronan solution from drying, and was placed in an oven at 40 °C for 16 h. During this time, the hyaluronan was cross-linked by the BDDE to form a gel.

#### Sodium periodate and chondroitinase treatments

A 10 mM solution of sodium (meta)periodate (Millipore Sigma, 71859-100G) was prepared in PBS, added to substrate wells, and incubated for 6 h. The reaction was then quenched with 0.1 M glycerol solution. At this point, the wells were washed three times with PBS and subsequently imaged. ChondroitinaseABC (Millipore Sigma, C3667-10UN) was aliquoted at 50 units ml^–1^ in PBS. Upon use, aliquots were diluted 1:15 in PBS and added to the treated wells. Samples were then incubated for 6 h, subsequently washed three times with PBS, and imaged. Coatings and gels were prepared in triplicates for both the untreated and treated condition. Collagen I, fibronectin, and laminin were treated with sodium periodate, whereas aggrecan was treated with ChondroitinaseABC. Three control wells were left with only PBS as controls. After treatment, all wells were incubated in a 5 µM Rhobo6 solution in PBS for 1 h and imaged using a confocal microscope. The fluorescence signal was quantified in each field of view as average intensity in a manually traced ROI containing the signal.

### Spectral imaging

A collagen I gel was prepared following methods described above, and was then incubated in 5 µM Rhobo6 solution in PBS for 1 h. An excitation scan was performed using a Leica Stellaris 8 confocal microscope, scanning excitation wavelengths from 500 nm to 566 nm in 2-nm increments. Emission was detected in the range of 575–630 nm. Spectra for ‘Collagen’ and ‘PBS’ were plotted averaging pixel value in manually traced ROIs. A spectral contrast image was generated by plotting the maximum absorbance wavelength in each pixel using MATLAB R2022a (MathWorks).

### Estimation of Rhobo6 binding affinity

Binding affinity to collagen type I was measured by the established method of fitting equilibrium constants^34^. Four collagen I gels (10 µl volume each) were prepared following the methods described above in a 50 mm glass-bottom Petri dish (MatTek, P50G-1.5-14-F). Directly after deposition of collagen solution on glass, a gentle tap was applied to distribute the gel on a larger surface, reducing its thickness. Upon use, each gel was equilibrated at room temperature in 2 ml of PBS for 60 min. Then, the gel was placed on a confocal microscope, and a focal plane within the collagen gel was established using brightfield contrast. The microscope was set up to acquire a 3-h time-lapse at a rate of 1 frame per min. A *t* = 0 time point was acquired before the addition of Rhobo6; then, 2 ml of Rhobo6 solution at twice the target concentration was added, resulting in a 1:1 dilution. The final concentration of Rhobo6 solution was altered for each of the four gels (1, 5, 25, 50 µM). After the acquisition, the mean intensity over the full field of view was extracted, and *k*_obs_ was determined by fitting the following equation in MATLAB R2022a (MathWorks):

$${{I}}({{t}})={{{I}}}_{\max }\left(1-{{\rm{e}}}^{\left(-{{{k}}}_{{\rm{obs}}}\left({{t}}-{{{t}}}_{0}\right)\right)}\right)+{{b}}$$

*I* is the measured intensity, *t* is the time, *b* is the background intensity, and *t*_0_ is a time delay parameter to take into account the arbitrary moment in which dye solution was added. Once all binding curves were acquired, a linear fit was performed between concentration and observed equilibrium constant following the relationship:

$${{{k}}}_{{\rm{obs}}}={{{k}}}_{{\rm{on}}}\left[{\rm{Rhobo}}6\right]+{{{k}}}_{{\rm{off}}}$$

*k*_on_ and *k*_off_ are the binding and unbinding constants, respectively. The *K*_d_ value for collagen type I was thus estimated as *K*_d_ = *k*_off_ / *k*_on_.

### Photobleaching test

A well in a glass-bottom eight-well plate (Ibidi, 80807) was coated with aggrecan, following the protocol described above. The coated glass was incubated with a 5 µM solution of Rhobo6 in PBS, with the well being completely filled with Rhobo6 solution and sealed with parafilm to prevent evaporation. The sample was imaged with a confocal microscope at 1 image per min overnight. The signal was plotted over time, calculated as the mean pixel value in manually traced ROIs.

### Mammalian cultured cell monolayer experiments

#### Cell culture conditions

Cells were maintained at 37 °C and 5% CO_2_. MCF10A GFP-MUC1∆CT cells (Paszek Lab, Cornell) were cultured in phenol-red-free 1:1 DMEM:F12 supplemented with 5% New Zealand horse serum (Thermo Fisher Scientific, 16050122), 20 ng ml^–1^ epidermal growth factor (Peprotech), 0.5 μg ml^–1^ hydrocortisone (Millipore Sigma, H0888-1G), 100 ng ml^–1^ cholera toxin (Millipore Sigma, C8052-.5MG), 10 μg ml^–1^ insulin (Millipore Sigma, I1882-100MG), and 1% penicillin–streptomycin (P/S) (Thermo Fisher Scientific, 15070063). PC-3 (ATCC, CRL-1435) cells were cultured in RPMI-1640 (Thermo Fisher Scientific,11875093) supplemented with 10% heat-inactivated fetal bovine serum (Thermo Fisher Scientific, 10-438-026) and 1% P/S.

#### Cell permeability experiment

PC-3 cells were plated in an 8-well plate (Ibidi, 80807) at 10,000 cells per well and cultured for 2 days. Rhobo or Rhobo6 were added at 5 µM through a 1:200 dilution from a 1 mM DMSO stock. Images were acquired on a confocal microscope upon addition (t = 0) and after 1, 2, and 6 h; cells were incubated at 37 °C and 5% CO_2_ between time points. The presence of intracellular dye was quantified as the average fluorescence signal within manually traced ROIs along cell perimeters for *n* = 9 cells for each condition and time point.

#### MCF10A GFP-MUC1∆CT experiments

Cells were plated at 10,000 cells per well in an 8-well dish (Ibidi, 80807) and cultured for 2 days. Doxycycline (Panreac AppliChem, A2951) was added 1 µg ml^–1^ through a 1:1,000 dilution from a 1 mg ml^–1^ stock in DMSO, and cells were incubated for another 2 days. Doxycycline induces the overexpression of the surface glycoprotein MUC1∆CT. Overexpression of MUC1∆CT causes cells to ball up and lift from their growth substrate, without loss of viability^35^. Once lifting was observed in the majority of cells, the wells were washed once with PBS, and the PBS was replaced with the indicated medium containing 5 µM Rhobo6. Fixation was performed with 4% paraformaldehyde (Electron Microscopy Sciences, 19202) in PBS for 30 min at room temperature, and mucinase treatment was performed with 100 nM StcE mucinase (expressed and purified as previously reported^36^) for 4 h at 37 °C.

### FLIM microscopy

MCF10A cells expressing GFP-MUC∆CT were plated and cultured as described above. Before imaging, cells were washed 3 times in PBS, and then incubated in a 5 µM Rhobo6 solution in PBS for 1 h under standard cell culture conditions (37 °C, 95% humidity, 5% CO_2_). FLIM microscopy was performed using an Abberior Facility Line microscope. Lifetime contrast images, phasor plot coordinates, and lifetime bandpass images were generated using the microscope software. The reported phasor plot was generated in MATLAB R2022a (Mathworks).

### Mouse embryonic salivary glands

#### Dissection and culture

All experiments complied with protocols approved by the Institutional Animal Care and Use Committee (IACUC) at Janelia Research Campus (protocol number 22-0230). Submandibular salivary glands (SMGs) were collected and cultured in vitro following the protocol reported by Wang et al.^38^. In brief, mouse submandibular salivary glands were dissected from embryos at E13–E14. The embryos were isolated from timed pregnant CD-1 outbred mice (Charles River Laboratories). Isolated salivary glands were cultured on 13-mm-diameter 0.1 µm pore polycarbonate filters (Millipore Sigma, WHA110405) floating on 1 ml organ culture medium (see below) in a 35-mm dish at 37 °C with 5% CO_2_. Organ culture medium was DMEM/F-12 (Thermo Fisher Scientific, 11039047) supplemented with 2 mM l-glutamine (Thermo Fisher Scientific, 25030081), 1× P/S (100 units ml^–1^ penicillin, 100 µg ml^–1^ streptomycin; Thermo Fisher Scientific, 15140163), 150 µg ml^–1^ vitamin C (Millipore Sigma, A7506), and 50 µg ml^–1^ transferrin (Millipore Sigma, T8158).

#### Rhobo6 incubation and mounting for imaging

For Rhobo6 dye labeling, 5 µl of 1 mM stock was diluted in 1 ml of organ culture medium to make a 5 µM labeling solution. Culture medium was replaced with the labeling solution, followed by 1 h of incubation at 37 °C with 5% CO_2_. To mount the samples for inverted microscope imaging, we used double-adhesive imaging spacers (Grace Bio-labs, 654002) attached to the 27-mm glass wide bottoms of 35-mm dishes (Thermo Fisher Scientific, 150682). Under a dissecting microscope, 5 µl organ culture medium was transferred to the center of the imaging spacer, and the filter with the glands was flipped onto the imaging spacer using a pair of forceps such that glands were sandwiched between the filter and the dish bottom. Care was taken to ensure the filter was flat and center-aligned with the imaging spacer. The edge of the filter was pressed to ensure tight adherence to the imaging spacer. Then, 1 ml organ culture medium with 5 µM Rhobo6 dye was added on top of the flipped filter. The mounted glands were imaged immediately.

#### Toxicity test during ex vivo culture

To evaluate whether Rhobo6 adversely affects the growth or branching morphogenesis of embryonic salivary glands cultured ex vivo, paired glands from the same embryo were separated into two groups, which were treated with 5 µM Rhobo6 (1 mM stock in DMSO) or 0.5% DMSO. Phase contrast images of cultured glands were acquired at 0, 24, and 48 h. The number of buds was counted manually on these images in Fiji using an ImageJ macro to facilitate recording of the results. To minimize bias, file names of all images were scrambled for observer blinding using a Python script before counting. The counting results were subsequently decoded and analyzed using customized Python scripts. The paired two-tailed *t*-test function from the SciPy package was used for pairwise comparison of the bud count between control and Rhobo6-treated groups.

#### Comparison of Rhobo and Rhobo6 labeling, along with washout

Salivary glands were cultured in organ culture medium for 4 days (see above). Two glands were incubated with Rhobo or Rhobo6 at 5 µM in organ culture medium, along with 2 µg ml^–1^ Hoechst 33342 (Thermo Fisher Scientific, 62249) to visualize nuclei, and Nucspot650 (Biotium, 41034-T) at a 1:500 dilution to visualize dead cells. After 2 h of incubation, glands were mounted as described above and imaged on a confocal microscope. Subsequently, samples were washed once with fresh warm medium and incubated at 37 °C with 5% CO_2_ for 15 min, then imaged on a confocal microscope. Two more washes with fresh warm medium were conducted during 180 min of total incubation time before being imaged a third and final time.

#### Antibody and protein labeling of live salivary glands

Anti-collagen-type-IV antibody (Sigma-Aldrich, AB769) and anti-laminin antibody (Sigma-Aldrich, L9393) were fluorescently labeled with Atto647N NHS ester (AAT Bioquest, 2856), following the manufacturer’s instructions. In brief, antibody vials were adjusted to pH ~8 through the addition of 1:20 vol/vol of 2 M sodium bicarbonate, pH 9. Atto647N NHS ester was dissolved to a concentration of 10 mM in anhydrous DMSO and incubated with each pH-corrected antibody at a 20:1 molar ratio for 1 h in the dark at room temperature. Free dye was removed using Zeba Spin Desalting Columns, 40K molecular weight cut-off, 0.5 ml (Thermo Fisher Scientific). Anti-laminin antibody from the manufacturer contained 1% wt/vol BSA as a stabilizer, which was fluorophore-labeled alongside the antibody, likely contributing to background signal in live salivary glands. CNA35-GFP was a gift from J. Northey (UCSF) and was expressed and purified as previously reported^40^. E13 submandibular salivary glands were isolated as described above and cultured for 2 days. Rhobo6 was added at 5 µM, alongside ~10 µg ml^–1^ of CNA35-GFP, anti-laminin antibody, or anti-collagen-type-IV antibody for 1 h. Imaging was performed in the staining solution without washing.

### Decellularized mouse tissues

Decellularized tissues were generated following the protocol reported by Narciso et al.^41^, with longer incubation times to account for thicker tissue sections. A 6-month-old C57BI/6J female mouse weighting 25 g was euthanized by cervical dislocation and dissected to extract whole heart and kidney. The organs were transferred to ice-cold HBSS (Thermo Fisher Scientific, 14-025-092). Organs were then cut in half, rinsed once more with HBSS, then glued on a vibratome support. To ensure stability during slicing, a drop of 2% low-melting-point agarose was used to gently coat the organs. Slices of 300 µm thickness for both organs were cut in ice-cold HBSS, then transferred to a dry 35-mm plastic dish and put in a −80 °C freezer. Two freeze–thaw cycles were performed, with freezing times of at least 6 h and thaw times of 2 h at room temperature. Then, slices were washed three times in PBS for 1 h at room temperature, followed by three 45-min baths in 2% sodium deoxycholate water solution at room temperature. After three more washes in PBS at 4 °C lasting 30 min each, slices were transferred into a solution of 0.5 mg ml^–1^ of DNAse I in 1 mM Tris-HCl solution containing 5 mM CaCl_2_ and 5 mM MgCl_2_. Slices were left in DNAse solution overnight at 4 °C. The following day, samples were washed three times in PBS for 30 min at room temperature and stored in PBS containing 1% P/S at 4 °C until use. For imaging, slices were incubated in PBS containing 5 µM Rhobo6 and 2 µg ml^–1^ Hoechst for 1 h at room temperature, transferred to a dry glass-bottom dish, and then imaged on a confocal microscope.

### Rhobo6 penetration in live tissue upon bathing

A 6-month-old C57BI/6J female mouse weighting 25 g was euthanized by cervical dislocation and dissected to extract whole quadricep muscle. The tissue sample was incubated in 3 ml of phenol-red-free RPMI-1640 medium (Innovative Research, IRPMI16400120500ML) supplemented with 20 mM HEPES and 5 µM Rhobo6 for 1 h at 37 °C, 95% humidity, and 5% CO_2_. Subsequently, the tissue was transferred to a dry plastic dish, and sliced along the longitudinal direction to reveal the inner cross section that was not in contact with the dye. The tissue was mounted on a dry 35-mm glass-bottom dish with a 27-mm window (Thermo Fisher Scientific, 150682) with the newly exposed cross section facing down, and was then imaged.

### Pancreatic tissue excised from mouse

#### Tissue collection

All experiments complied with protocols approved by the IACUC at Janelia Research Campus (protocol no. 16-142). C57BL/6J mice were obtained from the Jackson Laboratory. All surgical procedures were performed in animals under general anesthesia through administration of ketamine and xylazine (each 10 mg kg^–1^ body weight). Krebs-Ringer bicarbonate buffer (KRBH) containing 3 mM d-glucose was injected into the distally clamped bile duct using a 1 ml insulin syringe and a 31-gauge needle. The exsanguinated pancreas was then removed from the peritoneal cavity and cut into 1- to 5-mm pieces. Tissue pieces were placed in Krebs-Ringer bicarbonate buffer (KRBH) containing 3 mM d-glucose with 5 µM Rhobo6, 2 µg ml^–1^ Hoechst 33342 (Thermo Fisher Scientific, 62249), and/or 10 µg ml^–1^ Atto647N-anti-collagen-I (Novus Biologicals, NB600-408, fluorescently labeled as above). After 1 h of incubation at 37 °C, the tissue was mounted for imaging using double-adhesive imaging spacers and 35-mm glass-bottom dishes, as was done with salivary glands (see above).

#### STED microscopy

All experiments complied with protocols approved by the IACUC at Janelia Research Campus (protocol no. 22-0211). An 8-week-old C57BI/6J female mouse weighing 20 g was euthanized by cervical dislocation and dissected to extract pancreatic tissue. The tissue sample was cut into 1- to 5-mm pieces. The pieces were incubated in 3 ml RPMI-1640 phenol-red-free medium supplemented with 20 mM HEPES and 5 µM Rhobo6 for 1 h at 37 °C. To mount the tissue pieces stably for STED microscopy, samples were placed on a 35-mm glass-bottom dish (Thermo Fisher Scientific, 150682) and secured by a metal slice anchor (Warner Instruments, 64-1415); 500 µl of Rhobo6-containing medium was added on the sample to prevent drying. The mounted sample was imaged within 2 h using a Leica SP8 STED microscope with a 660 nm STED depletion laser. Both confocal and STED images of the same field of view were acquired for subsequential comparison. After acquisition, images were denoised (Supplementary Table 1) and an intensity plot was generated in Fiji/ImageJ by extracting pixel value along a line profile averaged over 10 pixels.

### Dye administration to mice and live-tissue imaging

All experiments complied with protocols approved by the IACUC at Janelia Research Campus (protocol number 22-0211). Mice used were 8- to 12-week-old C57BI/6J females (Jackson Laboratory) weighing between 20 and 22 g. Ten microliters of Rhobo6 solution at 10 mM in DMSO was diluted with sterile PBS to a 100 µl volume, yielding a 1 mM concentration. A mouse was transferred in an induction chamber and anesthetized with 2.5% isoflurane at an oxygen flow rate of 1.0 L min^–1^. All 100 µl of 1 mM Rhobo6 was then injected retro-orbitally using a 0.5-ml tuberculin syringe with a 27-gauge needle (BD, 305620), yielding a dose of ~3.5 mg kg^–1^ for a mouse weighing 20 g. Mice were allowed to recover in their cage over 30 min and were then euthanized by cervical dislocation. Tissues were dissected onto 35-mm glass-bottom dishes (Thermo Fisher Scientific, 150682). All tissues, except for trachea, were placed whole on the glass, for imaging through the fascia into the organ lumen. The trachea was mounted transversely, with the cross section facing the glass. Notably, mouse urine was bright pink within the 30-min recovery period before euthanasia, suggesting that the dye can be cleared through excretion.

#### Second-harmonic imaging and two-photon excitation autofluorescence microscopy

Jejenum, pancreas, and muscle tissues were collected following Rhobo6 retro-orbital injection, as described above. Once tissues were transferred to a 35-mm glass-bottom dish (Thermo Fisher Scientific, 150682), they were placed on a 2P microscope. Once a suitable field of view was identified, images of Rhobo6 fluorescence, 2P excitation autofluorescence microscopy autofluorescence, and second-harmonic generation were acquired (Supplementary Table 1). A control mouse (no Rhobo6 injection) was euthanized and dissected to collect the same tissues for comparison, which were mounted and imaged in a similar way.

### *C. elegans* husbandry and dye administration

Animals were reared at 20 °C on nematode growth medium (NGM) plates seeded with HB101 bacteria. Injections were performed at room temperature into the syncytium of the distal gonad arm of young to mid-adult N2 or NK2443 *[nid-1(qy38[nid-1::mNG+loxP]) V]* using standard procedures^62^. For Rhobo6 injections, dye aliquots were prepared through dilution with PBS to a final concentration of 100 µM dye and 1% DMSO and stored at −80 °C. Before injection, aliquots were thawed, centrifuged briefly for 5 min at 13,000*g*, and loaded into the microinjection needle. Animals were injected with approximately 10 pL of PBS or PBS with dye into each gonad arm. After injections, animals were rehydrated in M9 buffer and transferred to NGM plates seeded with HB101 to recover for 30–60 min. Injected animals were anaesthetized with 5 mM sodium azide and mounted on 2% agarose pads and imaged (Supplementary Table 1).

### *D. melanogaster* husbandry and dye administration

All flies in this study were raised at 25 °C with a 12-hour light–dark cycle. The following fly stocks were used (stock numbers are from the Bloomington Drosophila Stock Center): control (*w[1118]* (no. 3605)), neuronal cell driver (*w[1118]; P{y[+t7.7] w[+mC]=GMR57C10-GAL4}attP2* (no. 39171)) and the GFP fluorescent label (w[*]; P{y[+t7.7] w[+mC]=10XUAS-IVS-Syn21-GFP-p10}attP2)^63^. Fly brains were dissected in a chilled modified saline solution of 103 mM NaCl, 3 mM KCl, 5 mM TES, 8 mM trehalose, 10 mM glucose, 26 mm NaHCO_3_, 1 mM NaH_2_PO_4_, 2 mM CaCl_2_, and 4 mM MgCl_2_ at pH 7.4 and placed in a glass-bottom 8-well plate (Ibidi, 80807). The fly brains were oriented posterior side down on the plate. Once stuck to the base, the saline solution was removed and Rhobo6 was added at 5 µM. Brains were imaged within 1 h of dissection.

### *D. rerio* husbandry and dye administration

All experiments complied with protocols approved by the IACUC at Janelia Research Campus (protocol no. 22-0216). Larvae were reared at 28.5 °C in 14 h–10 h light–dark cycles. Zebrafish from 5 d.p.f. were fed rotifers, and were used for experiments at 8 d.p.f. Zebrafish sex cannot be determined until ~4 weeks after fertilization, so the sex of the animals was unknown. Fish were embedded in a drop of 2% low-melting-temperature agarose in a glass-bottom Petri dish. Agarose surrounding the tail was removed, and fish were anesthetized with MS-222 (0.16 mg ml^–1^). Water in the sample chamber was then replaced with Rhobo6 solution at 5 µM, diluted in tank water containing MS-222. To enable dye delivery, an incision in the tail fin was made using a tungsten needle (1-µm tip). Fish were incubated 30 min and then imaged.

### *A. thaliana* husbandry and dye administration

*Arabidopsis thaliana* seeds were obtained from the Arabidopsis Biological Resource Center (stock no. CS4004) and placed on a Lloyd & McCown Woody Plant Basal Medium with a Vitamins (PhytoTec Labs) agar pad sitting atop a 35-mm glass-bottom dish (Thermo Fisher Scientific, 150682). The pad and seeds were incubated at 4 °C for 3 days, then moved to room temperature under lights on the lab bench for 9 days. Rhobo6 was added at 5 µM to water surrounding the agar pad overnight. Root structures in the agar pad were imaged the following morning.

### 4T1 Spheroid invasion assay

#### Cell culture and spheroid generation

4T1 cells expressing mVenus fused to the transmembrane domain of MUC1 were a gift from the Weaver lab (UCSF). Spheroids were generated and embedded following established methods^64^. In brief, 4T1 cells were cultured in phenol-red-free RPMI medium (Innovative Research, IRPMI16400120500ML) supplemented with 10% heat-inactivated fetal bovine serum and 1% P/S. To generate spheroids with the hanging drop method, after trypsinization cells were resuspended in complete medium at 5,000 cells ml^–1^ and 20 droplets, each 20 µl, were deposited on the inside of the lid of a 10-cm Petri dish. After the lid was flipped onto a 10-cm Petri dish containing 10 ml warm PBS, the hanging droplets were incubated for 48 h at 37 °C and 5% CO_2_. Spheroids were collected in 200 µl complete medium and allowed to settle to the bottom of a 1.5-ml Eppendorf tube before embedding.

#### Spheroid embedding

Two hundred microliters of Growth Factor Reduced Matrigel (Corning, 354230) was added to a 24-well glass-bottom dish (MatTek, P24G-1.5-13-F) kept at 4 °C and then removed, to obtain a Matrigel coating. The coated plate was then incubated for 3 h at room temperature, then moved once again to 4 °C before the Matrigel dried. Collagen type I (Ibidi, 50201), received at 5 mg ml^–1^ from the manufacturer, was diluted to 4 mg ml^–1^ with 17.5 mM acetic acid, and 80-µl aliquots were stored at −20 °C. A DMEM solution was prepared by mixing 90 µl of 10x DMEM (Sigma, D2429), 7.5 µl of 1 M NaOH solution, 322.5 µl of water, and 30 µl of 7.5% of NaHCO_3_, then frozen in 80 µl stocks and stored at −20 °C. Growth Factor Reduced Matrigel was thawed at 4 °C, and made into 40-µl aliquots that were stored at −20 °C. To prepare an ECM mixture of 80% collagen type I at 2 mg ml^–1^ and 20% Growth Factor Reduced Matrigel, one of each aliquot was thawed at 4 °C then mixed, yielding a final volume of 200 µl. Two Eppendorf tubes containing 20 spheroids each were prepared as described above, and the bottom 20 µl of each was added to the 200 µl gel mixture, yielding a final volume of 240 µl. After mixing, 100 µl of the slurry was transferred to Matrigel-coated wells kept at 4 °C. In all steps, 200-µl pipette tips were widened by cutting off the bottom 2 mm using a razor blade, to avoid shear stress damage to spheroids. The plate was then moved to room temperature for 1 h, after which 1 ml of room temperature complete medium was added. The spheroids then moved to a 37 °C and 5% CO_2_ incubator.

#### Effect of Rhobo6 on spheroid invasive potential

Two sets of spheroids were generated and embedded as described above. After embedding, spheroids were bathed with medium containing 0.5% DMSO (vehicle) or with complete medium containing 5 µM Rhobo6 and 0.5% DMSO, with a total volume of 1 ml. After incubating the plate for 1 h at 37 °C, brightfield images were taken of spheroids in the two conditions every 24 h over 48 h (*n* = 16 spheroids per condition). The *t* = 0 h time point corresponded to approximately 2 h after embedding. The percentage invading area was calculated as (*A*_outer_ – *A*_inner_) / *A*_outer_, where *A*_inner_ is a manually traced region corresponding to the core spheroid area, and *A*_outer_ is a manually traced area of the entire spheroid, including all invading protrusions. Tracing was performed in Fiji/ImageJ. Statistical analysis was performed using Prism v.10.3.1 (GraphPad) through two-way ANOVA and Sidak’s multiple-comparisonstest, with the assumption of a single pooled variance.

#### Quantification of fiber orientation during spheroid invasion

Three spheroids were embedded as described above. After embedding, spheroids were bathed with complete medium containing 5 µM Rhobo6 and incubated for 1 h at 37 °C. Volumes of 553 μm × 553 μm × 162 μm were acquired once per hour over 2 days using an incubated 2P microscope (Supplementary Table 1). For time points, *t* = 0 corresponded to initial embedding in gel mixture. Fiji/ImageJ with the plugin OrientationJ^58^ was used for fiber orientation analysis. The Venus channel, which displayed spheroid cell membranes, was used to define rectangular ROIs in which the long side was parallel to invading protrusions. Highly-curved protrusions, those that protruded away from the plane of imaging, or those which protruded beyond the imaging area, were not analyzed. Next, a maximum intensity projection covering the full height of protruding cells was generated. Within each projection image containing an invading ROI, the Venus channel was used to draw a corresponding non-invading ROI in which the short side was parallel to the surface of the spheroid. For both types of ROI, the long side of the rectangle was defined as the 0° direction for orientation analysis. Intracellular regions in the Rhobo6 channel were masked. Then, for each ROI orientation in the Rhobo6 channel was determined per-pixel on the basis of the structure tensor using the OrientationJ ImageJ plugin^58^, with a Gaussian gradient and the following parameters: local window *σ* = 1, coherency threshold = 0%, energy threshold = 2%. Orientation distributions for *n* = 3 regions for each of *n* = 3 spheroids at the 24-h time point post-embedding were averaged. The reported s.d. was determined as root sum square of the s.d. of the ROIs in each spheroid.

### Intravital imaging of wild-type and MMTV-PyMT mice

#### Animals and animal care

Animal husbandry of mice was carried out in Laboratory Animal Resource Center (LARC) facilities at UCSF Parnassus in accordance with the guidelines stipulated by the IACUC protocol number AN194983, which adhere to the NIH Guide for the Care and Use of Laboratory Animals. Mice were maintained in pathogen-free, ventilated HEPA-filtered cages under stable housing conditions of 30–70% humidity, a temperature of 20–26 °C, and a 12–12 hour dark–light cycle. Ten-week-old female FVB/NJ and MMTV-PyMT mice on an FVB/NJ background were used for intravital imaging experiments.

#### Intravital imaging of the mammary gland

Intravital imaging of live animals was conducted according to the IACUC protocol number AN194983 within the Biological Imaging Development Center, which was approved by the UCSF IACUC for non-survival experiments. The procedure followed was similar to that described in Dawson et al.^65^. Before imaging, mice were anesthetized in a chamber using oxygen-delivered isoflurane gas (oxygen ~1 L min^–1^ flow rate and isoflurane vaporizer at ~3%), and Rhobo6 was quickly administered through retro-orbital injection before mice were allowed to recover for 15 min. Mice were then anesthetized as described above before they were transferred to a nose cone that was secured to a custom heated microscope stage attachment beneath the microscope objective. Depth of anesthesia was monitored by pedal or toe-pinch reflex and breathing rate and adjusted as needed throughout the imaging session. Two small midline incisions were made to open a flap of skin in the mouse which was gently pulled back to reveal the inguinal mammary gland for imaging. A custom metal annulus attached to the stage with height adjustable metal rods was positioned over the mammary gland and pressed to form a seal. A glass coverslip was then affixed to the annulus over the mammary gland with vacuum grease, and a drop of water was placed on the coverslip. The stage position was adjusted to place the imaging window directly below the objective lens.

#### Immunofluorescence of whole mounted mammary glands

The same mammary glands that were imaged by intravital microscopy (Fig. 5) were resected and fixed in 4% paraformaldehyde for 20 min. They were then washed with PBS, permeabilized with 0.3% Triton X-100 in PBS for 15 min before being blocked in PBS with 0.3% Triton X-100, 5% goat serum, and 3% bovine serum albumin for 1 h. Mammary glands were then incubated with CNA35-GFP and phalloidin-647 for 30 min to stain fibrillar collagens and filamentous actin, respectively, before being washed with PBS and incubated with DAPI for 10 min to stain nuclei. A final PBS wash was performed before glands were mounted with aquamount and a coverslip on a microscope slide. All incubations were done at room temperature, and small weights were placed over coverslips to flatten mounted mammary gland tissues as they dried overnight.

### Statistics and reproducibility

Replicates represent measurements taken from distinct samples, unless noted otherwise. Statistical methods were performed using Prism v.10.3.1 (GraphPad), unless noted otherwise. The statistical method used to analyze each figure subpanel is described in the corresponding figure legend. All images are representative of at least *n* = 2 independent replicates, except for the experiment shown in Figure 3d, which was performed one time.

### Reporting summary

Further information on research design is available in the Nature Portfolio Reporting Summary linked to this article.

## Online content

Any methods, additional references, Nature Portfolio reporting summaries, source data, extended data, supplementary information, acknowledgements, peer review information; details of author contributions and competing interests; and statements of data and code availability are available at 10.1038/s41592-024-02590-2.

## Supplementary information

Supplementary Information Supplementary Figure 1.

Reporting Summary

Supplementary Table 1 Microscopy methods, imaging parameters, and image-processing workflow for all microscopy data presented in the manuscript.

Supplementary Video 1 Time course of a coated aggrecan substrate incubated with Rhobo6, corresponding to Extended Data Figure 3b.

Supplementary Video 2 Time course of wound healing in zebrafish larvae incubated with Rhobo6, corresponding to Extended Data Figure 6e.

Supplementary Video 3 Three-dimensional render of Rhobo6-labeled blood vessel on brain surface, corresponding to Figure 4d (center).

Supplementary Video 4 Three-dimensional render of Rhobo6 labeled skin tissue, corresponding to Figure 4d (right).

Supplementary Video 5 2P microscopy time course of Rhobo6 labeled 4T1 spheroids expressing membrane tethered Venus and embedded in matrix, corresponding to Figure 5a.

## Source data

Source Data Statistical Source Data for Figs. 1–3 and 5.

Source Data Extended Data Figs. 1–5 and 8.

## Data Availability

Data supporting the findings of this study are available in the article. Unprocessed imaging datasets are available at a figshare repository: 10.6084/m9.figshare.25787688. Source data are provided with this paper.

## References

[1] Wu, D., Yamada, K. M. & Wang, S. Tissue morphogenesis through dynamic cell and matrix Interactions. *Annu. Rev. Cell Dev. Biol.* **39**, 123–144 (2023). 37315160 10.1146/annurev-cellbio-020223-031019 PMC11452922

[2] Chaudhuri, O., Cooper-White, J., Janmey, P. A., Mooney, D. J. & Shenoy, V. B. Effects of extracellular matrix viscoelasticity on cellular behaviour. *Nature* **584**, 535–546 (2020). 32848221 10.1038/s41586-020-2612-2 PMC7676152

[3] Nerger, B. A. et al. Local accumulation of extracellular matrix regulates global morphogenetic patterning in the developing mammary gland. *Curr. Biol.* **31**, 1903–1917 (2021). 33705716 10.1016/j.cub.2021.02.015 PMC8119325

[4] Hughes, A. J. et al. Engineered tissue folding by mechanical compaction of the mesenchyme. *Dev. Cell* **44**, 165–178 (2018). 29290586 10.1016/j.devcel.2017.12.004 PMC5826757

[5] Möckl, L. et al. Quantitative super-resolution microscopy of the mammalian glycocalyx. *Dev. Cell* **50**, 57–72 (2019). 31105009 10.1016/j.devcel.2019.04.035 PMC6675415

[6] Poole, J. J. A. & Mostaço-Guidolin, L. B. Optical microscopy and the extracellular matrix structure: a review. *Cells* **10**, 1760 (2021). 34359929 10.3390/cells10071760 PMC8308089

[7] Hu, M., Ling, Z. & Ren, X. Extracellular matrix dynamics: tracking in biological systems and their implications. *J. Biol. Eng.* **16**, 13 (2022). 35637526 10.1186/s13036-022-00292-x PMC9153193

[8] Isser, S. et al. Radiolabeled GPVI-Fc for PET imaging of multiple extracellular matrix fibers: a new look into pulmonary fibrosis progression. *J. Nucl. Med.* **64**, 940–945 (2023). 36702555 10.2967/jnumed.122.264552 PMC10241016

[9] Xenaki, K. T., Oliveira, S. & van Bergen en Henegouwen, P. M. P. Antibody or antibody fragments: implications for molecular imaging and targeted therapy of solid tumors. *Front. Immunol.* **8**, 1287 (2017). 29075266 10.3389/fimmu.2017.01287 PMC5643388

[10] Morgner, J. et al. A Lamb1Dendra2 mouse model identifies basement-membrane-producing origins and dynamics in PyMT breast tumors. *Dev. Cell* **58**, 535–549 (2023). 36905927 10.1016/j.devcel.2023.02.017

[11] Jones, R. A. et al. An mTurq2-Col4a1 mouse model allows for live visualization of mammalian basement membrane development. *J. Cell Biol.* **223**, e202309074 (2023). 38051393 10.1083/jcb.202309074 PMC10697824

[12] Chen, X., Nadiarynkh, O., Plotnikov, S. & Campagnola, P. J. Secondharmonic generation microscopy for quantitative analysis of collagen fibrillar structure. *Nat. Protoc.* **7**, 654–669 (2012). 22402635 10.1038/nprot.2012.009 PMC4337962

[13] Junqueira, L. C., Cossermelli, W. & Brentani, R. Differential staining of collagens type I, II and III by Sirius Red and polarization microscopy. *Arch. Histol. Jpn.* **41**, 267–274 (1978). 82432 10.1679/aohc1950.41.267

[14] Biela, E. et al. Col-F, a fluorescent probe for ex vivo confocal imaging of collagen and elastin in animal tissues. *Cytom. A* **83A**, 533–539 (2013). 10.1002/cyto.a.22264 PMC3671577 23404939

[15] Karamanos, N. K. et al. A guide to the composition and functions of the extracellular matrix. *FEBS J.* **288**, 6850–6912 (2021). 33605520 10.1111/febs.15776

[16] Griffin, M. E. & Hsieh-Wilson, L. C. Tools for mammalian glycoscience research. *Cell* **185**, 2657–2677 (2022). 35809571 10.1016/j.cell.2022.06.016 PMC9339253

[17] Cheng, B., Tang, Q., Zhang, C. & Chen, X. Glycan labeling and analysis in cells and in vivo. *Annu. Rev. Anal. Chem.* **14**, 363–387 (2021). 10.1146/annurev-anchem-091620-091314 34314224

[18] Lopez Aguilar, A. et al. Tools for studying glycans: recent advances in chemoenzymatic glycan labeling. *ACS Chem. Biol.* **12**, 611–621 (2017). 28301937 10.1021/acschembio.6b01089 PMC5469623

[19] Zeng, Y., Ramya, T. N. C., Dirksen, A., Dawson, P. E. & Paulson, J. C. High-efficiency labeling of sialylated glycoproteins on living cells. *Nat. Methods* **6**, 207–209 (2009). 19234450 10.1038/nmeth.1305 PMC2830088

[20] Albertazzi, L. & Heilemann, M. When weak is strong: a plea for low-affinity binders for optical microscopy. *Angew. Chem. Int. Ed.* **62**, e202303390 (2023). 10.1002/anie.202303390 37158582

[21] Bucevičius, J., Lukinavičius, G. & Gerasimaitė, R. The use of Hoechst dyes for DNA staining and beyond. *Chemosensors* **6**, 18 (2018).

[22] Graham, B. J., Windsor, I. W., Gold, B. & Raines, R. T. Boronic acid with high oxidative stability and utility in biological contexts. *Proc. Natl Acad. Sci.**USA* **118**, e2013691118 (2021). 33653951 10.1073/pnas.2013691118 PMC7958229

[23] Li, D., Chen, Y. & Liu, Z. Boronate affinity materials for separation and molecular recognition: structure, properties and applications. *Chem. Soc. Rev.* **44**, 8097–8123 (2015). 26377373 10.1039/c5cs00013k

[24] Fang, G. et al. Recent development of boronic acid-based fluorescent sensors. *RSC Adv.* **8**, 29400–29427 (2018). 35548017 10.1039/c8ra04503h PMC9084483

[25] Williams, G. T., Kedge, J. L. & Fossey, J. S. Molecular boronic acid-based saccharide sensors. *ACS Sens.* **6**, 1508–1528 (2021). 33844515 10.1021/acssensors.1c00462 PMC8155662

[26] Sun, X., Zhai, W., Fossey, J. S. & James, T. D. Boronic acids for fluorescence imaging of carbohydrates. *Chem. Commun.* **52**, 3456–3469 (2016). 10.1039/c5cc08633g 26728041

[27] Kim, K. K. et al. Postcolumn HPLC detection of mono- and oligosaccharides with a chemosensor. *Org. Lett.* **5**, 5007–5010 (2003). 14682751 10.1021/ol035978q PMC3376175

[28] Sibrian-Vazquez, M., Escobedo, J. O., Lowry, M. & Strongin, R. M. Progress toward red and near-infrared (NIR) emitting saccharide sensors. *Pure Appl. Chem.* **84**, 2443–2456 (2012). 23504507 10.1351/PAC-CON-11-11-06 PMC3596891

[29] Halo, T. L., Appelbaum, J., Hobert, E. M., Balkin, D. M. & Schepartz, A. Selective recognition of protein tetraserine motifs with a cell-permeable, pro-fluorescent bis-boronic acid. *J. Am. Chem. Soc.* **131**, 438–439 (2009). 19105691 10.1021/ja807872s PMC2659559

[30] James, T. D. & Shinkai, S. in *Host-Guest Chemistry: Mimetic Approaches to Study Carbohydrate Recognition* 1st edn (ed. Penadés, S.) 159–200 (Springer, 2002).

[31] Sun, X. et al. The mechanisms of boronate ester formation and fluorescent turn-on in ortho-aminomethylphenylboronic acids. *Nat. Chem.* **11**, 768–778 (2019). 31444486 10.1038/s41557-019-0314-x PMC8573735

[32] Tønnesen, J., Inavalli, V. V. G. K. & Nägerl, U. V. Super-resolution imaging of the extracellular space in living brain tissue. *Cell* **172**, 1108–1121 (2018). 29474910 10.1016/j.cell.2018.02.007

[33] Grimm, J. B. & Lavis, L. D. Synthesis of rhodamines from fluoresceins using Pd-catalyzed C–N cross-coupling. *Org. Lett.* **13**, 6354–6357 (2011). 22091952 10.1021/ol202618t PMC3235915

[34] Jarmoskaite, I., AlSadhan, I., Vaidyanathan, P. P. & Herschlag, D. How to measure and evaluate binding affinities. *eLife* **9**, e57264 (2020). 32758356 10.7554/eLife.57264 PMC7452723

[35] Shurer, C. R. et al. Physical principles of membrane shape regulation by the glycocalyx. *Cell* **177**, 1757–1770.e21 (2019). 31056282 10.1016/j.cell.2019.04.017 PMC6768631

[36] Pedram, K., et al. Design of a mucin-selective protease for targeted degradation of cancer-associated mucins. *Nat. Biotechnol*. **42**, 597–607 (2023). 10.1038/s41587-023-01840-6 PMC11018308 37537499

[37] Zhang, X.-F., Zhang, Y. & Liu, L. Fluorescence lifetimes and quantum yields of ten rhodamine derivatives: structural effect on emission mechanism in different solvents. *J. Lumin.* **145**, 448–453 (2014).

[38] Wang, S., Matsumoto, K., Lish, S. R., Cartagena-Rivera, A. X. & Yamada, K. M. Budding epithelial morphogenesis driven by cell-matrix versus cell–cell adhesion. *Cell* **184**, 3702–3716 (2021). 34133940 10.1016/j.cell.2021.05.015 PMC8287763

[39] Velicky, P. et al. Dense 4D nanoscale reconstruction of living brain tissue. *Nat. Methods* **20**, 1256–1265 (2023). 10.1038/s41592-023-01936-6 PMC10406607 37429995

[40] Aper, S. J. A. et al. Colorful protein-based fluorescent probes for collagen imaging. *PLoS ONE* **9**, e114983 (2014). 25490719 10.1371/journal.pone.0114983 PMC4260915

[41] Narciso, M. et al. Novel decellularization method for tissue slices. *Front. Bioeng. Biotechnol.* **10**, 832178 (2022). 35356779 10.3389/fbioe.2022.832178 PMC8959585

[42] Lukinavičius, G. et al. Fluorogenic probes for live-cell imaging of the cytoskeleton. *Nat. Methods* **11**, 731–733 (2014). 24859753 10.1038/nmeth.2972

[43] Laughlin, S. T. & Bertozzi, C. R. In vivo imaging of *Caenorhabditis elegans* glycans. *ACS Chem. Biol.* **4**, 1068–1072 (2009). 19954190 10.1021/cb900254y PMC2807738

[44] Keeley, D. P. et al. Comprehensive endogenous tagging of basement membrane components reveals dynamic movement within the matrix scaffolding. *Dev. Cell* **54**, 60–74 (2020). 32585132 10.1016/j.devcel.2020.05.022 PMC7394237

[45] Hoogenboom, J. et al. Direct imaging of glycans in *Arabidopsis* roots via click labeling of metabolically incorporated azido-monosaccharides. *BMC Plant Biol.* **16**, 220 (2016). 27724898 10.1186/s12870-016-0907-0 PMC5056477

[46] Acerbi, I. et al. Human breast cancer invasion and aggression correlates with ECM stiffening and immune cell infiltration. *Integr. Biol.* **7**, 1120–1134 (2015). 10.1039/c5ib00040h PMC4593730 25959051

[47] Maller, O. et al. Tumour-associated macrophages drive stromal cell-dependent collagen crosslinking and stiffening to promote breast cancer aggression. *Nat. Mater.* **20**, 548–559 (2021). 33257795 10.1038/s41563-020-00849-5 PMC8005404

[48] Northey, J. J., Przybyla, L. & Weaver, V. M. Tissue force programs cell fate and tumor aggression. *Cancer Discov.* **7**, 1224–1237 (2017). 29038232 10.1158/2159-8290.CD-16-0733 PMC5679454

[49] Ray, A. & Provenzano, P. P. Aligned forces: origins and mechanisms of cancer dissemination guided by extracellular matrix architecture. *Curr. Opin. Cell Biol.* **72**, 63–71 (2021). 34186415 10.1016/j.ceb.2021.05.004 PMC8530881

[50] Lin, E. Y. et al. Progression to malignancy in the polyoma middle T oncoprotein mouse breast cancer model provides a reliable model for human diseases. *Am. J. Pathol.* **163**, 2113–2126 (2003). 14578209 10.1016/S0002-9440(10)63568-7 PMC1892434

[51] Grimm, J. B. et al. A general method to optimize and functionalize red-shifted rhodamine dyes. *Nat. Methods* **17**, 815–821 (2020). 32719532 10.1038/s41592-020-0909-6 PMC7396317

[52] Sandanayake, K. R. A. S., Imazu, S., James, T. D., Mikami, M. & Shinkai, S. Molecular fluorescence sensor for saccharides based on amino coumarin. *Chem. Lett.* **24**, 139–140 (1995).

[53] Riera, R. et al. Single-molecule imaging of glycan–lectin interactions on cells with Glyco-PAINT. *Nat. Chem. Biol.* **17**, 1281–1288 (2021). 34764473 10.1038/s41589-021-00896-2

[54] Schnitzbauer, J., Strauss, M. T., Schlichthaerle, T., Schueder, F. & Jungmann, R. Super-resolution microscopy with DNA-PAINT. *Nat. Protoc.* **12**, 1198–1228 (2017). 28518172 10.1038/nprot.2017.024

[55] Moen, E. et al. Deep learning for cellular image analysis. *Nat. Methods* **16**, 1233–1246 (2019). 31133758 10.1038/s41592-019-0403-1 PMC8759575

[56] Bloom, W. & Fawcett, D. W. *A Textbook of Histology* (Chapman & Hall, 1994).

[57] Treuting, P. M., Dintzis, S. M. & Montine, K. S. (eds) *C**omparative Anatomy and Histology: A Mouse, Rat, and Human Atlas* 2nd edn (Academic Press, 2017).

[58] Fonck, E. et al. Effect of aging on elastin functionality in human cerebral arteries. *Stroke* **40**, 2552–2556 (2009). 19478233 10.1161/STROKEAHA.108.528091

[59] Lim, S., Escobedo, J. O., Lowry, M. & Strongin, R. M. Detecting specific saccharides via a single indicator. *Chem. Commun.* **47**, 8295 (2011). 10.1039/c1cc11343g PMC3377375 21643594

[60] Mütze, J. et al. Excitation spectra and brightness optimization of two-photon excited probes. *Biophys. J.* **102**, 934–944 (2012). 22385865 10.1016/j.bpj.2011.12.056 PMC3283774

[61] Makarov, N. S., Drobizhev, M. & Rebane, A. Two-photon absorption standards in the 550–1600 nm excitation wavelength range. *Opt. Express* **16**, 4029–4047 (2008). 18542501 10.1364/oe.16.004029

[62] Rieckher, M. & Tavernarakis, N. Generation of *Caenorhabditis elegans* transgenic animals by DNA microinjection. *Bio. Protoc.* **7**, e2565 (2017). 29071286 10.21769/BioProtoc.2565 PMC5734614

[63] Pfeiffer, B. D., Truman, J. W. & Rubin, G. M. Using translational enhancers to increase transgene expression in *Drosophila*. *Proc. Natl Acad. Sci. USA* **109**, 6626–6631 (2012). 22493255 10.1073/pnas.1204520109 PMC3340069

[64] Berens, E. B., Holy, J. M., Riegel, A. T. & Wellstein, A. A cancer cell spheroid assay to assess invasion in a 3D setting. *J. Vis. Exp.* **20**, 53409 (2015). 10.3791/53409 PMC4692745 26649463

[65] Dawson, C. A., Mueller, S. N., Lindeman, G. J., Rios, A. C. & Visvader, J. E. Intravital microscopy of dynamic single-cell behavior in mouse mammary tissue. *Nat. Protoc.* **16**, 1907–1935 (2021). 33627843 10.1038/s41596-020-00473-2

