# Supplementary material for: Live imaging of the extracellular matrix with a glycan-binding fluorophore
Source: Nat Methods. 2025 Feb 6;22(5):1070–80. doi: 10.1038/s41592-024-02590-2 (PMC12074998; doi:10.1038/s41592-024-02590-2)
Supplement: Supplementary file 1 — Supplementary Figure 1. [file 41592_2024_2590_MOESM1_ESM.pdf]

nature methods

Supplementary information https://doi.org/10.1038/s41592-024-02590-2

Live imaging of the extracellular matrix with a glycan-binding fluorophore

In the format provided by the authors and unedited

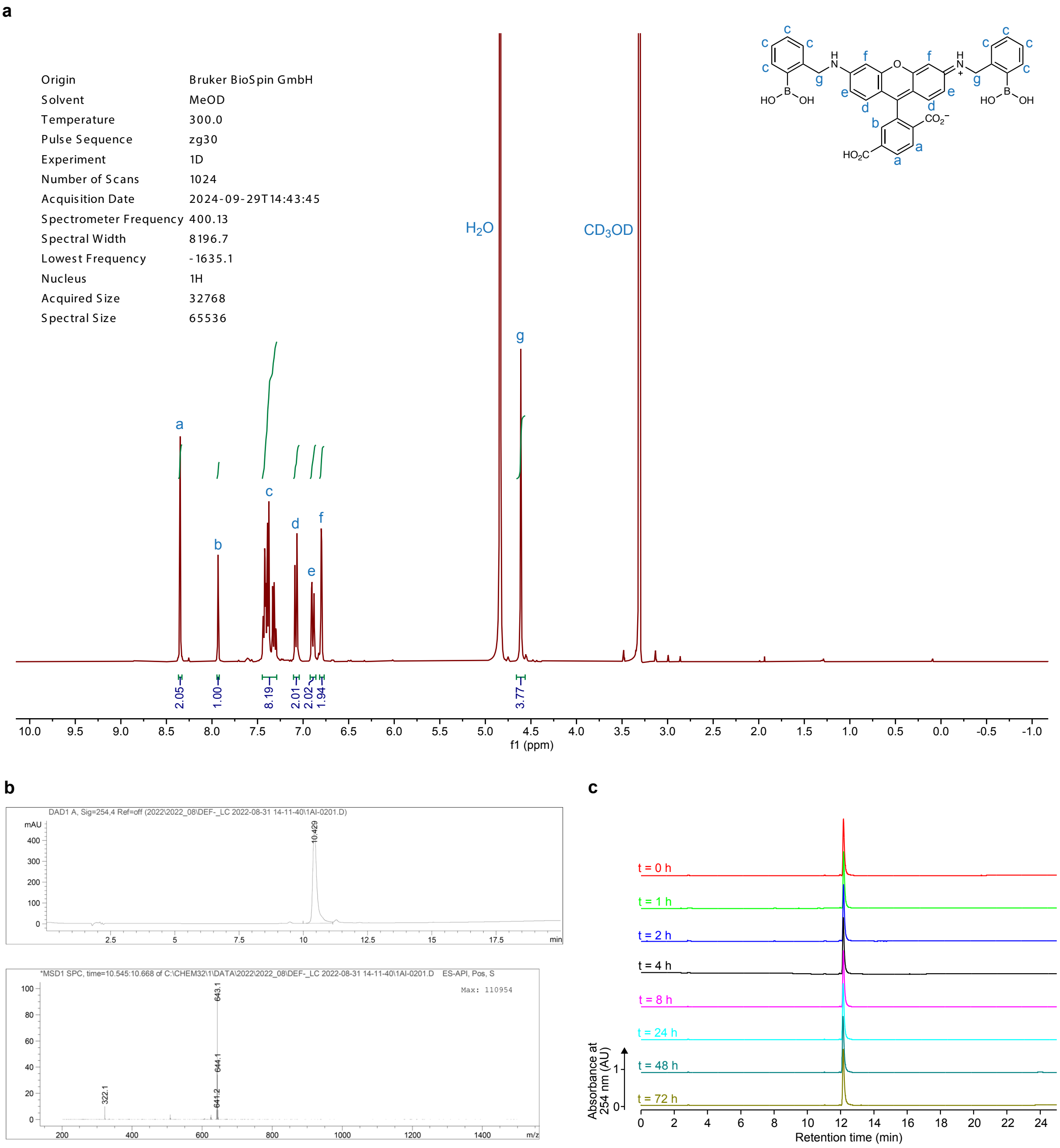

Supplementary Figure 1. Chemical characterization of Rhobo6.
a, ^{1}H NMR spectrum of Rhobo6. **b,** Analytical liquid chromatography mass spectrometry trace with absorbance detection at 254 nm (*top*) and mass spectrum of main peak (*bottom*). **c**, Stability of Rhobo6 at room temperature in 1:1 DMSO:PBS over time, as assessed by HPLC with absorbance detection at 254 nm. Calculated Rhobo6 purity ranged from 95-96% over the 72 h period.
