# Supplementary material for: Live imaging of the extracellular matrix with a glycan-binding fluorophore
Source: Nat Methods. 2025 Feb 6;22(5):1070–80. doi: 10.1038/s41592-024-02590-2 (PMC12074998; doi:10.1038/s41592-024-02590-2)
Supplement: Supplementary file 2 — Reporting Summary [file 41592_2024_2590_MOESM2_ESM.pdf]

nature portfolio

Corresponding author(s): Kayvon Pedram

Last updated by author(s): Nov 5, 2024

Reporting Summary

Nature Portfolio wishes to improve the reproducibility of the work that we publish. This form provides structure for consistency and transparency in reporting. For further information on Nature Portfolio policies, see our Editorial Policies and the Editorial Policy Checklist.

Statistics

For all statistical analyses, confirm that the following items are present in the figure legend, table legend, main text, or Methods section.

n/a	Confirmed	
☐	☒	The exact sample size (*n*) for each experimental group/condition, given as a discrete number and unit of measurement
☐	☒	A statement on whether measurements were taken from distinct samples or whether the same sample was measured repeatedly
☐	☒	The statistical test(s) used AND whether they are one- or two-sided<br>*Only common tests should be described solely by name; describe more complex techniques in the Methods section.*
☒	☐	A description of all covariates tested
☐	☒	A description of any assumptions or corrections, such as tests of normality and adjustment for multiple comparisons
☐	☒	A full description of the statistical parameters including central tendency (e.g. means) or other basic estimates (e.g. regression coefficient) AND variation (e.g. standard deviation) or associated estimates of uncertainty (e.g. confidence intervals)
☐	☒	For null hypothesis testing, the test statistic (e.g. *F*, *t*, *r*) with confidence intervals, effect sizes, degrees of freedom and *P* value noted<br>*Give P values as exact values whenever suitable.*
☒	☐	For Bayesian analysis, information on the choice of priors and Markov chain Monte Carlo settings
☒	☐	For hierarchical and complex designs, identification of the appropriate level for tests and full reporting of outcomes
☒	☐	Estimates of effect sizes (e.g. Cohen's *d*, Pearson's *r*), indicating how they were calculated

Our web collection on statistics for biologists contains articles on many of the points above.

Software and code

Policy information about availability of computer code

Data collection	Leica Application Suite X v.4.7.0 (Stellaris8 platform) or v.3.5.7 (SP8 platform); Zeiss Zen Blue v.2.3; Nikon NIS Elements v.5.42.03; Abberior Imspector v.16.3.
Data analysis	Fiji/ImageJ x64 (NIH) v.1.54j; MATLAB (Mathworks) v. R2022a; Prism (GraphPad) v. 10.3.1; Nikon NIS Elements v.5.42.03.

For manuscripts utilizing custom algorithms or software that are central to the research but not yet described in published literature, software must be made available to editors and reviewers. We strongly encourage code deposition in a community repository (e.g. GitHub). See the Nature Portfolio guidelines for submitting code & software for further information.

Data

Policy information about availability of data

All manuscripts must include a data availability statement. This statement should provide the following information, where applicable:

- Accession codes, unique identifiers, or web links for publicly available datasets
- A description of any restrictions on data availability
- For clinical datasets or third party data, please ensure that the statement adheres to our policy

Data supporting the findings of this study are available within the article. Unprocessed imaging datasets are available at the figshare repository: https://doi.org/10.6084/m9.figshare.25787688.

Research involving human participants, their data, or biological material

Policy information about studies with human participants or human data. See also policy information about sex, gender (identity/presentation), and sexual orientation and race, ethnicity and racism.

Reporting on sex and gender	n/a
Reporting on race, ethnicity, or other socially relevant groupings	n/a
Population characteristics	n/a
Recruitment	n/a
Ethics oversight	n/a

Note that full information on the approval of the study protocol must also be provided in the manuscript.

Field-specific reporting

Please select the one below that is the best fit for your research. If you are not sure, read the appropriate sections before making your selection.

☒ Life sciences ☐ Behavioural & social sciences ☐ Ecological, evolutionary & environmental sciences

For a reference copy of the document with all sections, see nature.com/documents/nr-reporting-summary-flat.pdf

Life sciences study design

All studies must disclose on these points even when the disclosure is negative.

Sample size	Sample size for each experiment is reported in the corresponding figure caption.
Data exclusions	No data were excluded.
Replication	The number of replicates and statistical tests for each experiment are reported in the corresponding figure caption or the Statistics and Reproducibility section.
Randomization	All samples were randomly allocated into experimental groups.
Blinding	For assessment of salivary gland growth and morphogenesis, file names of all images were scrambled for observer blinding before bud counting.

Reporting for specific materials, systems and methods

We require information from authors about some types of materials, experimental systems and methods used in many studies. Here, indicate whether each material, system or method listed is relevant to your study. If you are not sure if a list item applies to your research, read the appropriate section before selecting a response.

Materials & experimental systems

n/a	Involved in the study	
☐	☒	Antibodies
☐	☒	Eukaryotic cell lines
☒	☐	Palaeontology and archaeology
☐	☒	Animals and other organisms
☒	☐	Clinical data
☒	☐	Dual use research of concern
☐	☒	Plants

Methods

n/a	Involved in the study	
☒	☐	ChIP-seq
☒	☐	Flow cytometry
☒	☐	MRI-based neuroimaging

Antibodies

Antibodies used	Anti-collagen I (Novus Biologicals, NB600-408), Anti-Collagen Type IV antibody (Sigma-Aldrich, AB769), Anti-Laminin antibody (Sigma-Aldrich, L9393)

Validation	Anti-Collagen I antibody was validated by the manufacturer for ELISA, Western Blot, ICC/IF, IHC, IP, and microarray in human, mouse, rat, porcine, bovine, and rabbit samples. Anti-Collagen Type IV was validated by the manufacturer for ELISA, dot blot, ICC/IF, and IHC in human, bovine, and mouse samples. Anti-Laminin was validated by the manufacturer for dot blot, IHC, and microarray in human and mouse samples.

Eukaryotic cell lines

Policy information about cell lines and Sex and Gender in Research

Cell line source(s)	PC-3 (ATCC, CRL-1435), MCF10A + GFP-MUC1ΔCT (Paszek Lab, Cornell), 4T1-membraneVenus (Weaver Lab, UCSF)
Authentication	Cell lines were not authenticated.
Mycoplasma contamination	All cell lines were negative for mycoplasma.
Commonly misidentified lines (See ICLAC register)	No commonly misidentified cell lines were used in this study.

Animals and other research organisms

Policy information about studies involving animals; ARRIVE guidelines recommended for reporting animal research, and Sex and Gender in Research

Laboratory animals	Mouse (CD-1, 13-14 day old embryos; C57Bl/6J, 2-6 month old females; FVB/NJ and MMTV-PyMT on FVB/NJ background, 10 week old females)<br>C. Elegans (N2 or NK2443 [nid-1(qy38[nid-1::mNG+loxP]) V], young to mid-adult)<br>D. Melanogaster (Control (w[1118] (#3605)), neuronal cell driver (w[1118]; P{y[+t7.7] w[+mC]=GMR57C10-GAL4}attP2 (#39171)) and the GFP fluorescent label (w[*]; P{y[+t7.7] w[+mC]= 10XUAS-IVS-Syn21-GFP-p10}attP2, 3-5 days old females)<br>D. Rerio (Casper, 8 d.p.f larvae)
Wild animals	No wild animals were used in this study.
Reporting on sex	For breast cancer models, females were used. Tissues were collected from female mice.
Field-collected samples	No field collected samples were used in this study.
Ethics oversight	All experiments complied with protocols approved by the Institutional Animal Care and Use Committee (IACUC) at Janelia Research Campus and UCSF.

Note that full information on the approval of the study protocol must also be provided in the manuscript.

Dual use research of concern

Policy information about dual use research of concern

Hazards

Could the accidental, deliberate or reckless misuse of agents or technologies generated in the work, or the application of information presented in the manuscript, pose a threat to:

No	Yes	
☒	☐	Public health
☒	☐	National security
☒	☐	Crops and/or livestock
☒	☐	Ecosystems
☒	☐	Any other significant area

Experiments of concern

Does the work involve any of these experiments of concern:

No	Yes	
☒	☐	Demonstrate how to render a vaccine ineffective
☒	☐	Confer resistance to therapeutically useful antibiotics or antiviral agents
☒	☐	Enhance the virulence of a pathogen or render a nonpathogen virulent
☒	☐	Increase transmissibility of a pathogen
☒	☐	Alter the host range of a pathogen
☒	☐	Enable evasion of diagnostic/detection modalities
☒	☐	Enable the weaponization of a biological agent or toxin
☒	☐	Any other potentially harmful combination of experiments and agents

Plants

Seed stocks	Arabidopsis thaliana seeds were obtained from the Arabidopsis Biological Resource Center (stock no. CS4004).
Novel plant genotypes	n/a
Authentication	n/a
